# Mineral crude drug mirabilite (Mangxiao) inhibits the occurrence of colorectal cancer by regulating the *Lactobacillus*–bile acid–intestinal farnesoid X receptor axis based on multiomics integration analysis

**DOI:** 10.1002/mco2.556

**Published:** 2024-04-25

**Authors:** Xiaohang Zhou, Hui Sun, Junling Ren, Guangli Yan, Le Yang, Honglian Zhang, Haitao Lu, Xinghua Li, Toshiaki Makino, Fengting Yin, Jing Li, Xijun Wang

**Affiliations:** ^1^ State Key Laboratory of Integration and Innovation of Classical Formula and Modern Chinese Medicine National Chinmedomics Research Center National TCM Key Laboratory of Serum Pharmacochemistry Metabolomics Laboratory Department of Pharmaceutical Analysis Heilongjiang University of Chinese Medicine Harbin China; ^2^ State Key Laboratory of Dampness Syndrome The Second Affiliated Hospital Guangzhou University of Chinese Medicine Guangzhou China; ^3^ Department of Traditional Chinese Medicine, Pharmacy College Qiqihar Medical University Qiqihar China; ^4^ Hong Kong Traditional Chinese Medicine Phenome Research Centre, School of Chinese Medicine Hong Kong Baptist University Hong Kong China; ^5^ State Key Laboratory of Environmental and Biological Analysis Hong Kong Baptist University Hong Kong China; ^6^ Department of Pharmacognosy Graduate School of Pharmaceutical Sciences Nagoya City University Nagoya Japan

**Keywords:** colorectal cancer, Mangxiao, multiomics, traditional Chinese medicine

## Abstract

Mineral crude drug has revolutionized the treatment landscape in precision oncology niche that leads to the improvement in therapeutic efficiency on various tumor subtypes. Mangxiao (MX), a mineral crude drug in traditional Chinese medicine, has been used for treating gastrointestinal diseases for thousands of years. However, the action mechanisms are still ambiguous. Here, we attempt to explore inhibitory roles and associated pharmacological mechanisms of MX upon colorectal cancer (CRC) in APC^Min/+^ male mice by integrating metabolomics, 16S rDNA sequencing analyses, and metagenomic‐based microbiota analysis. We found that MX can significantly inhibit the occurrence of CRC through the regulation of the dysregulated gut microbe metabolism. Furthermore, the correlation analysis of metabolomes and 16S rDNA revealed that MX could restore the disorders of gut microbes by specifically enriching the abundance of Lactobacilli to improve bile acid metabolism, which further activated the farnesoid X receptor (FXR) in CRC mice, then the improvement of gut dysbiosis could inhibit the development of CRC. Collectively, our effort confirmed MX has the capacity to intervene the development of CRC and further discovered that it targets *Lactobacillus*–bile acid–intestinal FXR axis, which can be regarded as a candidate medicine for future drug discovery and development against CRC.

## Graphical abstract

We found that Mangxiao (MX) was a unique mineral medicine that was effective in prevention and cure colorectal cancer. It was first time to find that MX could reshape the *Lactobacillus*–bile acid–FXR axis, thereby inhibiting the occurrence and development of CRC. The bacterial‐targeted approach to control CRC provided a basis for the development of unique anticolorectal cancer drugs.

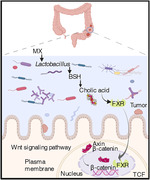

## 1 INTRODUCTION

Mineral drugs have been used clinically for the treatment of multiple complex diseases for thousands of years in China. In recent years, many studies primarily focus on the pharmacological exploration of mineral drugs on cancer treatment,^1^, ^2^ microcirculation improvement,^3^ gastrointestinal regulation,^4^ and so on. Basically, the priority roles of mineral drugs on the intervention of the occurrence and development of many tumors, for example, Arsenic (As_2_O_3_), were observed to exert significantly therapeutic efficiency against hematological tumors, including acute promyelocytic leukemia (APL),^5^, ^6^ as this mineral drug could improve the therapeutic outcome of APL patients at all development stages and has been approved by the United States Food and Drug Administration (US FDA) for the clinical treatment of APL.^7^ Realgar, a mineral crude drug with the main component of arsenic sulfide (As_4_S_4_), had significant inhibitory effect on human cancer cell line^8^ such as human uterine cervix cancer cell^9^ and lung cancer stem cell.^10^ Mangxiao (MX) is another famous purgative mineral crude drug that was a crystalline substance purified from mirabilite containing mainly hydrated sodium sulfate Na_2_SO_4_▪10H_2_O, which was first recorded in traditional Chinese medicine (TCM) classical book “Miscellaneous Records of Famous Physicians (Mingyi Bielu)” published in Han Dynasty. It has been used to treat gastrointestinal tumors and associated complications,^11^ and in our previous study, we found MX could effectively treat colorectal cancer (CRC) by modulating systems‐metabolism network.^12^, ^13^ These results suggested that MX can act as a mineral agent for the CRC treatment and demonstrated a great application value. However, unclear in‐depth mechanism of the therapeutic effects greatly restricted its further clinical and widespread usage.

Intestinal adenoma was considered to be the main precancerous lesion of CRC, and about 80% of CRC cases evolved from intestinal adenoma.^14^ CRC was one of the common malignant tumors occurred within digestive tract, posing a serious threat to human health with about 1.1 million new cases and 0.6 million new deaths worldwide in 2020.^15^ According to the American Cancer Society Report, CRC was the second most common cause of cancer death in the United States. In 2023, approximately 153,020 individuals would be diagnosed with CRC and 52,550 would die from the disease.^16^, ^17^ From an epidemiological perspective, the occurrence of colorectal adenomas is closely associated with genetic factors, social environment, diet, and other lifestyle habits. Excessive alcohol consumption, smoking, consumption of large amounts of red meat, reduced intake of dietary fiber, obesity, lack of physical exercise, and so on also increased the risk of developing colorectal adenomas.^18^

Beyond our understanding of the interactions of genetics and environments driven CRC development,^19^ there is increasing evidence to show that the occurrence of intestinal cancer was closely associated with metabolic disorders of gut microbes.^20^, ^21^, ^22^ *Escherichia coli* and enterotoxigenic *Bacteroides fragilis* can cause the changes of intestinal microenvironments to initiate local inflammation and subsequent cellular DNA damage, thus sparing inducing the formation of intestinal tumors and exacerbating tumor deterioration.^23^ Besides, abnormalities in gut microbiota can affect multiple‐stages progression of CRC progression, including promoting cancer cell proliferation and tumor immune escape.^24^, ^25^, ^26^ In addition to exert the influence on cell microorganisms, gut microbiota and its metabolites also induced intestinal diseases, for example, gut microbiota‐derived metabolic pathway, particularly microbial bile acid (BA) metabolism, played important roles in CRC progression.^27^

In the present study, we proposed that MX could inhibit the occurrence of CRC by improving the dysregulated gut microbes and associated metabolism. We used APC^Min/+^ mice as an intestinal tumor model created by genome editing technology with the mutation of adenomatosis polyposis coli gene,^28^ and our data demonstrated that MX could potently reduce the numbers and the size of intestinal tumors, as well as prevent pathological malignant transformation of intestinal tumors. An integrative multiomics approach with gut‐microbiota 16S rDNA sequencing technology and metagenomic‐based microbiota analysis and metabolomics method were employed to profoundly investigate the pharmacological mechanism of MX against CRC. We also validated the reshaping effect of MX on the gut microbiota in the treatment experiment. Moreover, the differences in the mechanism between farnesoid X receptor (FXR) agonist and MX were compared (Figure S1). These data indicated MX could greatly enrich the abundance of Lactobacilli to improve the dysregulated BA metabolism by activating FXR signaling pathway in CRC mice. In short, our research verifies MX is a novel active drug for intervening CRC through *Lactobacillus*–BA–intestinal FXR axis to regulate dysregulated metabolism in CRC.

## 2 RESULTS

### 2.1 MX intervention significantly inhibits the formation of intestinal adenoma in APC^Min/+^ mice

**FIGURE 1:**
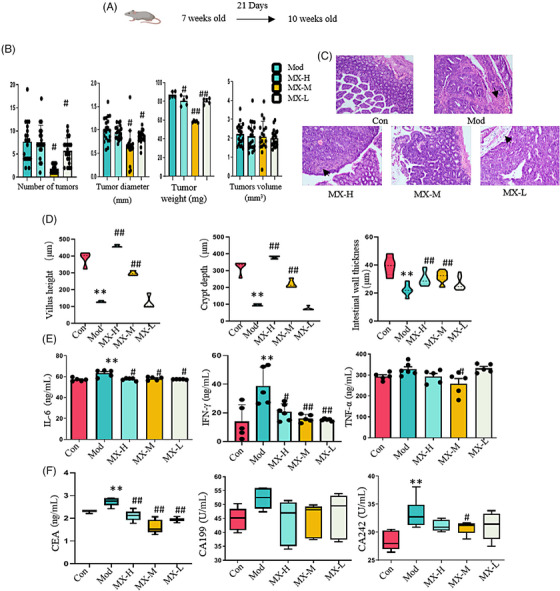
MX influenced the growth of intestinal tumors. (A) Transgenic mice were given MX since 7 weeks old for 21 days. (B) The comparison of the number of tumors, tumor diameter, weight, and volume among model (Mod) and MX‐treated groups with different doses (MX‐H, 6 g/kg/d; MX‐M, 4 g/kg/d; and MX‐L, 2 g/kg/d). Control group (Con) did not exhibit any tumors. (C) Representative histopathological observation of the intestine. (D) The comparison of the villus height, crypt depth, and intestinal wall thickness in the histopathological evaluation. (E) The comparison of the levels of IL‐6, IL‐10, IFN‐γ, and TNF‐α in serum. (F) The comparison of the levels of CEA, CA199, and CA242 in serum. ^*^ *p* < 0.05, ^**^ *p* < 0.01 versus control group; ^#^ *p* < 0.05, ^##^ *p* < 0.01 versus model group.

To investigate whether MX can exert an antitumor effect, we designed a 3‐week intervention study in APC^Min/+^mice (Figure 1A). The number of tumors was significantly reduced in MX‐treated groups, specially the average number of tumors in middle‐dosage group of MX intervention, and was 1.8 (inhibition rate of 75.6%) with tumors diameter being 0.62 ± 0.34 mm (inhibition rate of 38%); in contrast, the number was 7.4 and the diameter was 1.00 ± 0.26 mm in APC^Min/+^ model group. The average weight of tumors was 87.0 ± 3.3 mg in the model group, while MX could reduce the average weight of tumors with the weight of 58.4 ± 1.1 mg (inhibition rate of 32.9%) (Figure 1B). Moreover, the details of hematoxylin and eosin (H&E) staining results showed that the intestinal morphology change and inflammatory lesions were alleviated in the groups treated with MX (Figure 1C), and the villus height, crypt depth, and intestinal wall thickness were also significantly increased in MX groups (Figure 1D). The levels of inflammatory cytokines of interleukin (IL)‐6, interferon (IFN)‐γ, and tumor necrosis factor (TNF)‐α in serum can be regulated by the treatment with MX (Figure 1E), so did the tumor biomarker levels of carcinoembryonic antigen (CEA), carbohydrate antigen (CA) 199, and CA242 in the group treated with MX (Figure 1F). Different doses of MX‐treated groups exhibited appreciable antitumor effects, especially the middle dose group. The above data implied MX exhibited robust efficacy against tumor in spontaneous intestinal adenoma mice, and middle dose group was also selected as the optimal dose for the remaining experiments.

### 2.2 MX affected host metabolism under intervention experiment

**FIGURE 2:**
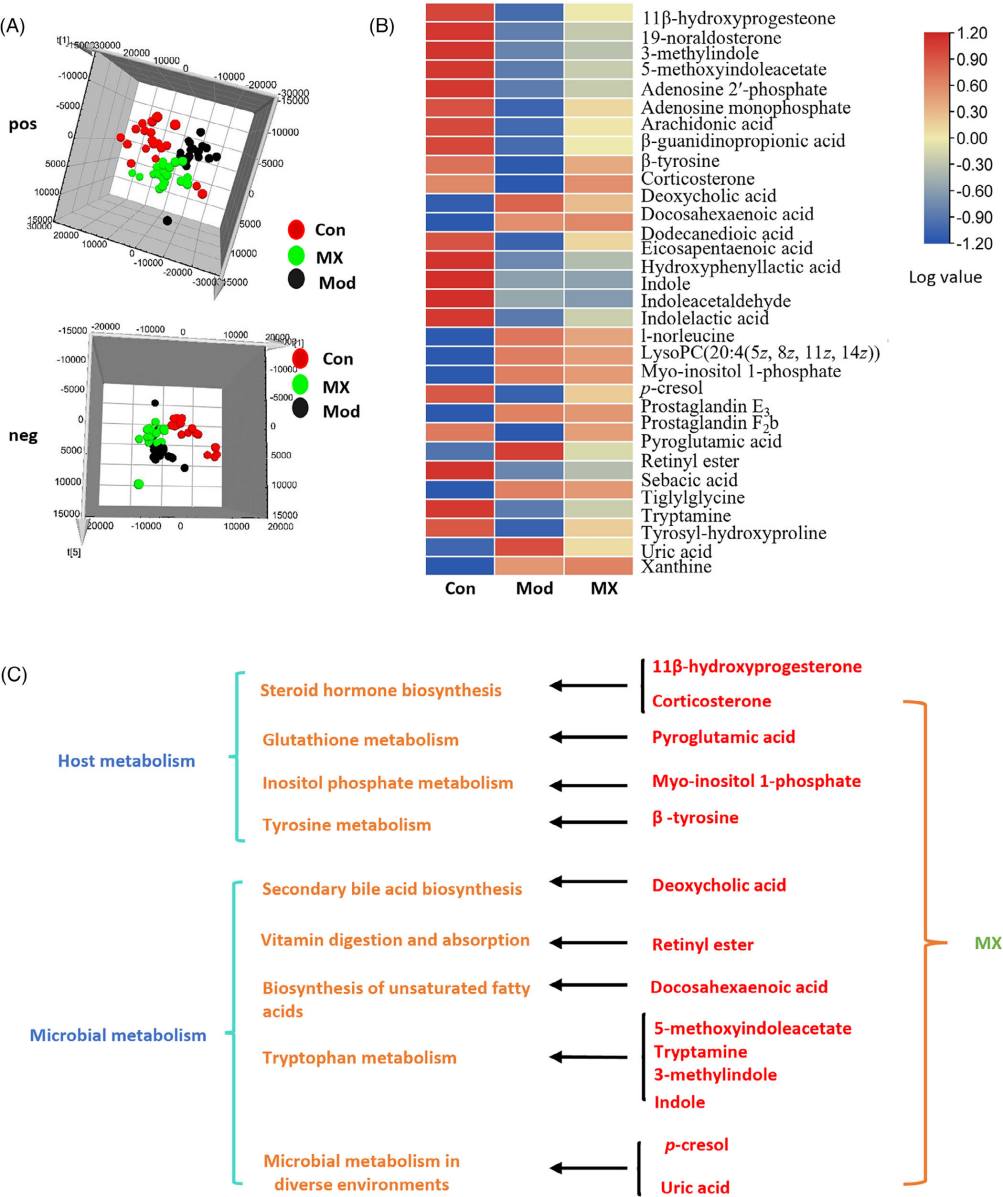
Effect of MX on host metabolism. (A) PCA score plots of MX regulating host serum metabolism in positive (pos) and negative (neg) ion mode. (B) Heatmap analysis of untargeted metabolomics of serum metabolism among control group (Con), model group (Mod), and MX‐treated group (MX, 4 g/kg/d) in CRC development. (C) Metabolic pathway analysis of pharmacodynamic metabolites.

On the basis of understanding mechanisms for the inhibitory effect of MX on intestinal adenomas, its impact on the host from the perspective of metabolic phenotype were investigated. Untargeted serum metabolomics was performed after the intervention of MX. Partial least squares‐discriminant (PLS‐DA) analysis showed that the control, model, and MX‐treated groups loaded in different spatial distributions, indicating a significant difference among three groups in serum metabolism (Figure 2A). Thirty metabolites were significantly changed in APC^Min/+^mice, and MX was able to regulate the disturbance of 27 of that in serum metabolism (Figure 2B and Tables S1 and S2). Based on the enrichment of the differentially expressed metabolites by KEGG, these metabolites could be clustered into two groups: microbiome‐derived metabolites and host metabolites. Moreover, the vast majority of metabolic pathways were related to microorganisms (Figure 2C).

### 2.3 MX mediated microbiota‐modulated metabolites under intervention experiment

**FIGURE 3:**
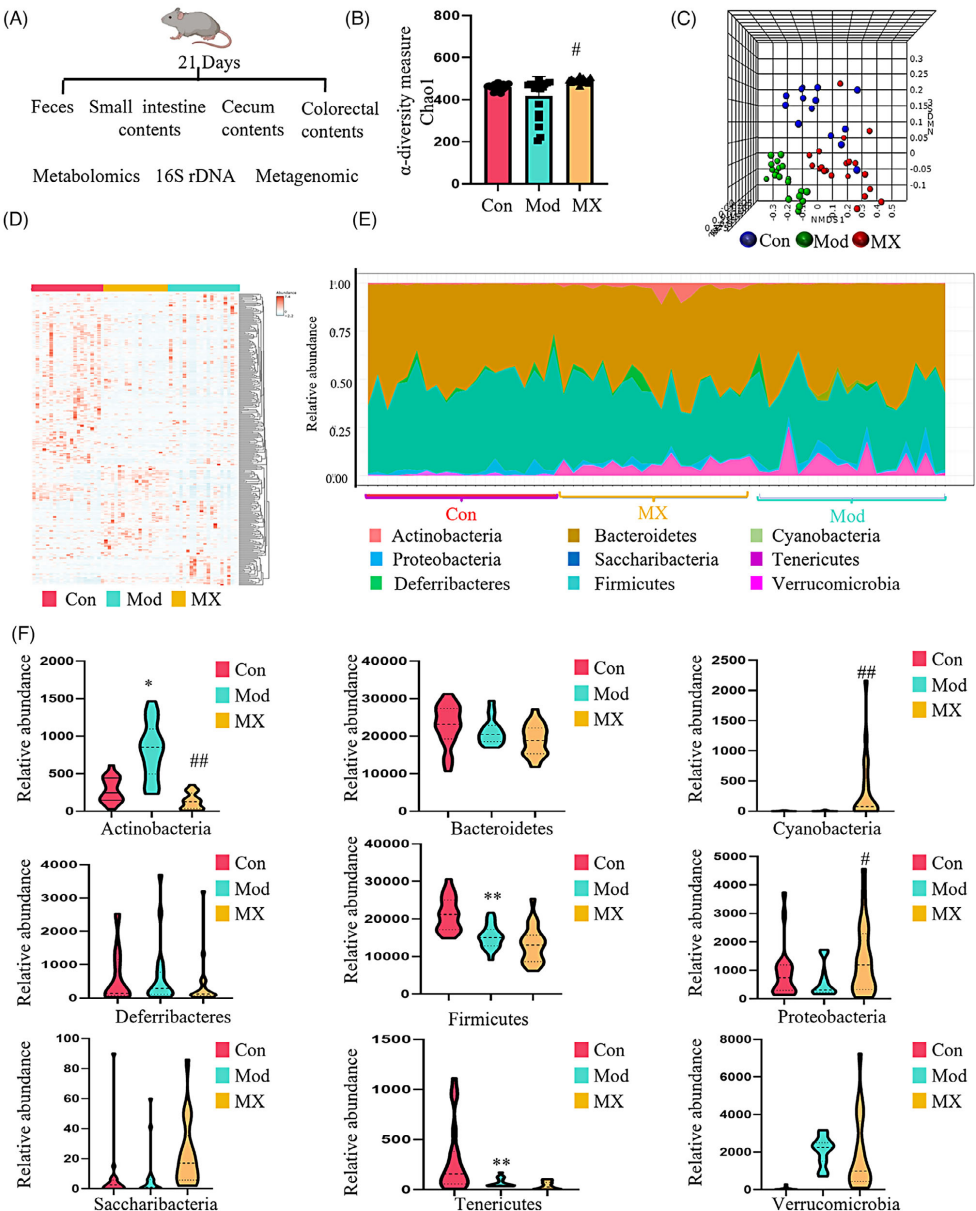
Effect of MX on microbiota‐modulated metabolites as potential mediator during the occurrence of intestinal tumor. (A) After 21 days of MX intervention on intestinal adenoma, metabolomics analysis was performed on intestinal contents, cecal contents, colorectal contents, and fecal samples, and then intestinal microflora analysis was performed on collected fecal samples. (B) The comparison of α‐diversity among control group (Con), model group (Mod), and MX‐treated group (MX, 4 g/kg/d). (C) The comparison of β‐diversity. (D) The cluster heatmap of microbial operation taxonomic units. (E) Area plot showing average percentage of bacterial population in Phylum level. (F) The relative abundance of bacteria in Phylum level.

**FIGURE 4:**
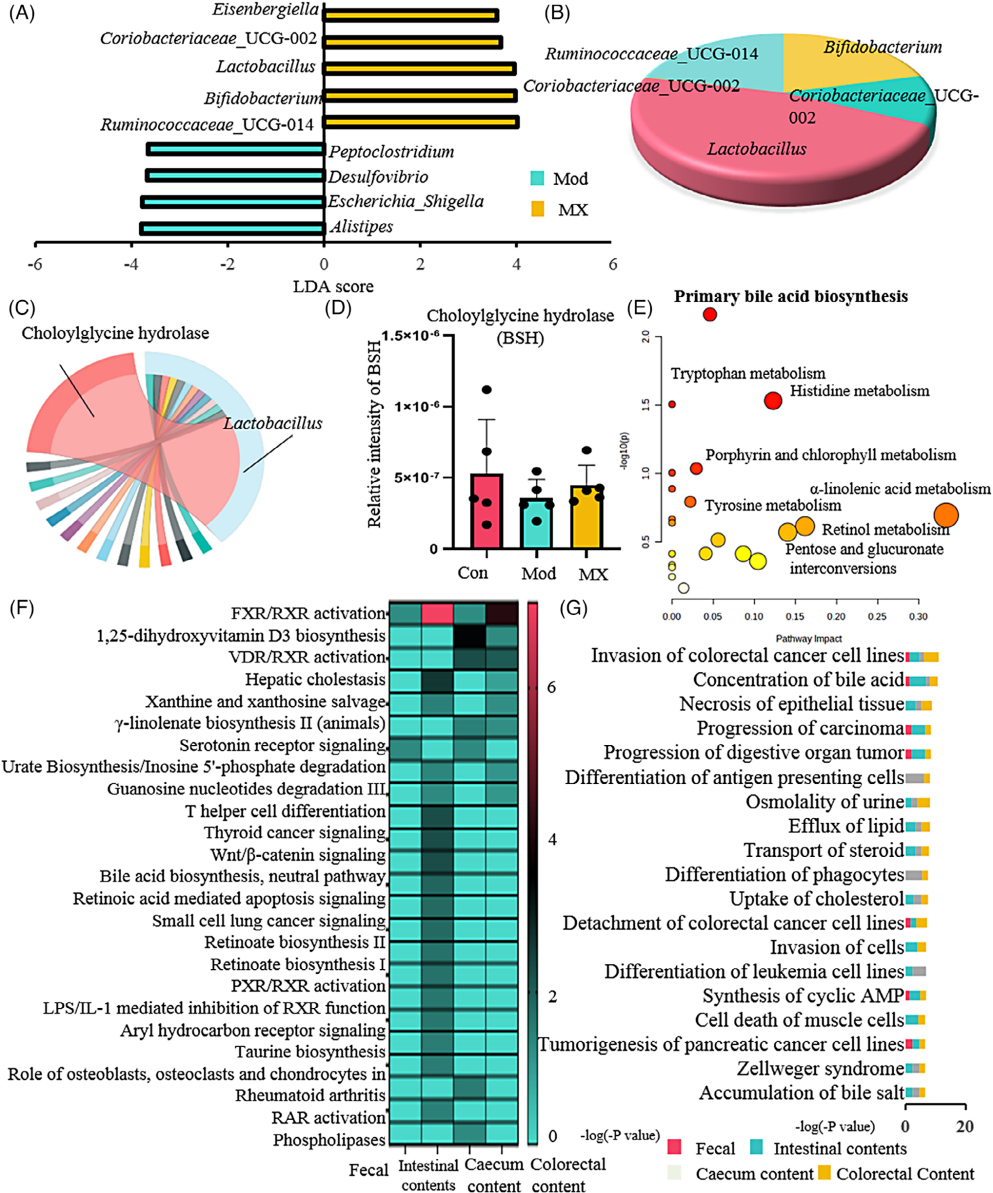
Effect of MX on microbiota‐modulated metabolites as potential mediator during the occurrence of intestinal tumor. (A) Histogram of the LDA scores computed for features differentially abundant among control and MX‐treated groups. Only taxa meets an LDA significant threshold > 3.5 and *p* < 0.05 were shown. (B) Pie chart of the proportion of each key microorganism. (C) Chord of the choloylglycine hydrolase (BSH, EC3.5.1.24, KO1442) affected by MX in *Lactobacillus*. (D) The relative intensity of BSH among control group (Con), model group (Mod), and MX‐treated group (MX, 4 g/kg/d). (E) Metabolic pathway enrichment analysis of differentia metabolites. (F) and (G) Event and disease predictions using detected metabolites in each tissue content. *p < 0.05, **p < 0.01 versus control group; ^#^ *p* < 0.05, and ^##^ *p* < 0.01 versus model group.

In order to investigate whether the intervention effect of MX depended on the gut microbiota, the overall structural changes of gut microbiota in response to MX were determined by the analysis of the 16S rDNA gene sequences of microbial samples isolated from the feces of control, model and MX‐treated groups (Figure 3A). The results of α‐diversity analysis demonstrated MX could improve the diversity and richness of gut microbes compared with model group (Figure 3B). In order to evaluate distinct clustering of gut microbes for each group, the principal coordinate analysis score plot was established. The gut microbes in MX‐treated group were far away from the model group and closed to the control group, and the same results could be obtained from the cluster heatmap of microbial operation taxonomic units (Figures 3C and D), which indicated that MX‐induced microbial composition changes under the action of intervention effect. Further, the taxonomic analysis of microorganisms at phylum, class, order, family, and genus level was carried out, and Actinobacteria, Bacteroidetes, Firmicutes, and Proteobacteria were the dominant microorganisms at phylum level (Figure 3E). Taxonomic profiles among three groups were first compared at phylum level and revealed that relative abundances of them were closer to the control group after administration of MX (Figures 3E and F). Next, to identify the specific genera biomarkers associated with MX's effect, a cladogram was used to describe the structure of the gut microbiota and the predominant bacteria between model and MX‐treated groups. All taxa identified by linear discriminant analysis effect size (LEfSe) exceed a linear discriminant analysis (LDA) score of 3.5, which indicated significant differences between groups (Figure 4A). LEfSe analyses showed that nine of taxa were differentially abundant in control and MX‐treated groups. In these taxa, *Coriobacteriaceae_UCG‐002*, *Lactobacillus*, *Bifidobacterium*, *Ruminococcaceae_UCG‐014*, and *Eisenbergiella* were enriched in MX‐treated group, of which, *Lactobacillus* were the most related microbes in high abundance and intervention effect (Figure 4B). This suggests that *Lactobacillus* may be a key gut microbiota for MX to intervene the occurrence stage of intestinal adenoma. The genetic abundance of *Lactobacillus* in the control and model groups was further analyzed, and the top 30 genes from differential genes between two groups were selected (Figure 4C). Choloylglycine hydrolase (BSH, EC3.5.1.24, KO1442) was the most differentially expressed genes in *Lactobacillus* between the control and model groups during the tumorigenesis (although this difference has no statistical significance), and MX had a promoting effect on its expression (Figure 4D). These enzymes are produced by the gut microbiota and catalyze the deconjugation of glycine‐ or taurine‐conjugated BAs, and thus increasing BSH could contribute to the increased concentrations of unconjugated BAs.

To assess metabolic alternations of gut microbiota remodeled by MX, untargeted metabolomic strategy was performed to explore the profile of intestine contents and feces by ultraperformance liquid chromatography–electrospray ionization quadrupole time‐of‐flight–mass spectrometry (UPLC–QTOF–MS). Significant differences including numbers and abundance of peaks, as well as the metabolomic profiles of the intestine and feces existed between control and MX‐treated groups (Figures S2 and S3). The metabolic trajectory was also altered after the intervention of MX (Figure S4). In addition, principal component analysis (PCA) showed that two groups loaded in spatial distribution within different clustering, which indicated that there was a significant difference in the endogenous metabolism (Figures S5 and S6). Compared with the model group, MX could reverse the abnormal expression of most metabolites in intestinal contents, caecum content, colorectal content, and fecal, such as cholic acid, taurocholic acid, and so on (Figures S7–S10 and Tables S3–S14). Based on MetaboAnalyst, the topology map was generated to describe the effects of MX on these responsive metabolites, BA synthesis was regarded as the most significant pathway (Figure 4E). Furthermore, we used the ingenuity pathway analysis (IPA) platform to interpret possible targets of MX. The canonical pathways analysis of the related metabolites showed that FXR/retinoid X receptor (RXR) activation were strongly correlated with MX efficacy (Figures 4F and G). It also confirmed the analysis of the metabolic pathways modulated by MX in the MetPA analysis. These results suggested that MX influenced BA metabolism, thereby activating the FXR receptor, and finally played a therapeutic role in the intestinal tumor.

### 2.4 MX protected mice against intestinal tumor under treatment experiment

**FIGURE 5:**
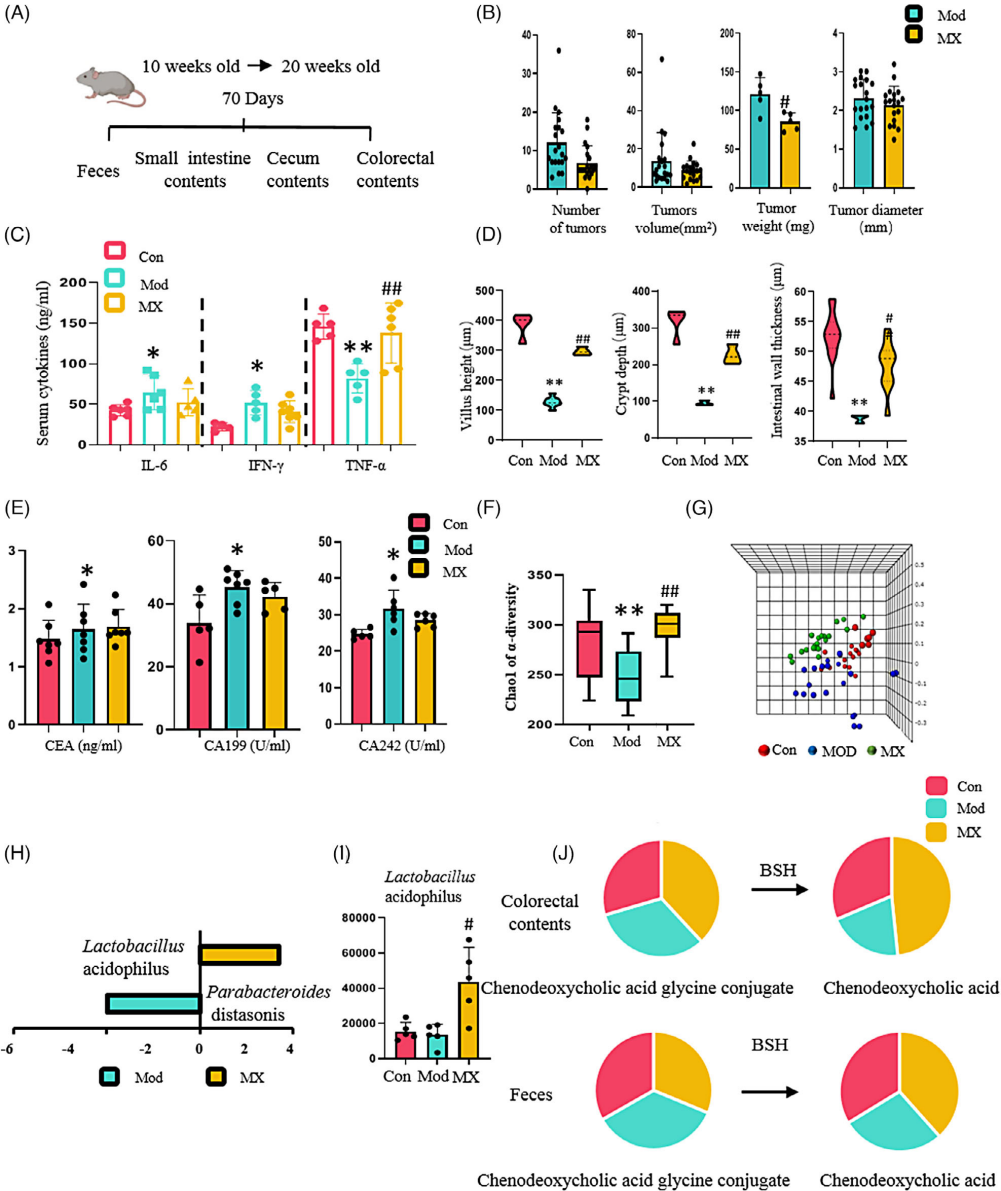
MX influenced the microenvironment of intestinal tumors. (A) Transgenic mice were given MX since 10 weeks old for 70 days. (B) The comparison of the number of tumors, adenoma volume, weight, and diameter between model group and MX group. The comparison of the number of tumors, tumor diameter, weight, and volume among model group (Mod), and MX‐treated group (MX, 4 g/kg/d). Control group (Con) did not exhibit any tumors. (C) The comparison of the serum levels of IL‐6, IFN‐γ, and TNF‐α. (D) The comparison of the villus height, crypt depth, and intestinal wall thickness. (E) The comparison of serum level of the CEA, CA199, and CA242. (F) The comparison of α‐diversity. (G) The comparison of β‐diversity. (H) Histogram of the LDA scores computed for features differentially abundant among control group (Con) and MX‐treated group (MX, 4 g/kg/d). Only taxa meet an LDA significant threshold > 3.5 and *p* < 0.05 were shown. (I) The comparison of the *Lactobacillus* acidophilus. (J) BSH contributed to the increased concentrations of unconjugated bile acids from glycine‐conjugated bile acids in colorectal contents and fecal samples.

To investigate the efficacy of MX on CRC, the treatment experiment lasted for 10 weeks was conducted (Figure 5A). The administration of MX significantly reduced the weight of intestinal tumors (Figure 5B), the expression of inflammatory factors (Figure 5C), and prevented pathological malignant transformation of intestinal tumors (Figures 5D and E). Thus, these results showed that MX had a certain therapeutic effect on the development of intestinal tumor, abnormal inflammatory factors, and deterioration of intestinal tumors. Consistent with the intervene effects on gut microbes, MX greatly enhanced the diversity of gut microbes and made it close to control group (Figures 5F and G). Furthermore, metagenome LEfSe analysis of key microorganisms was carried out (Figure 5H). The results also showed that *Lactobacillus* was the key genus‐level microorganism under the treatment of MX, and *L. acidophilus* was the key species (Figure 5I), which was consistent with the intervention effect of MX. Then, we performed targeted metabolomics profiling of the BAs level in feces and intestinal contents from different groups by UPLC–QTOF–MS. In colorectal contents and fecal samples, the relative intensity of chenodeoxycholic acid was increased by BSH (Figure 5J). Consistently, the levels of BAs were significantly changed in feces and intestinal contents of model group compared with those of control group, while MX treatment reversed these changes (Figure S11A). To confirm the target of MX regulation, we imported these BAs into KEGG and IPA online software for metabolic pathway enrichment analysis. The results revealed that these metabolites were most related with invasion of CRC cell lines and the canonical pathways analysis was also related to FXR/RXR activation (Figures S11B and S11C). These data suggest that MX protected mice against intestinal tumor through gut microbiota and further affected BA–FXR axis.

### 2.5 MX influenced the metabolic intestinal flora profiles of health host

**FIGURE 6:**
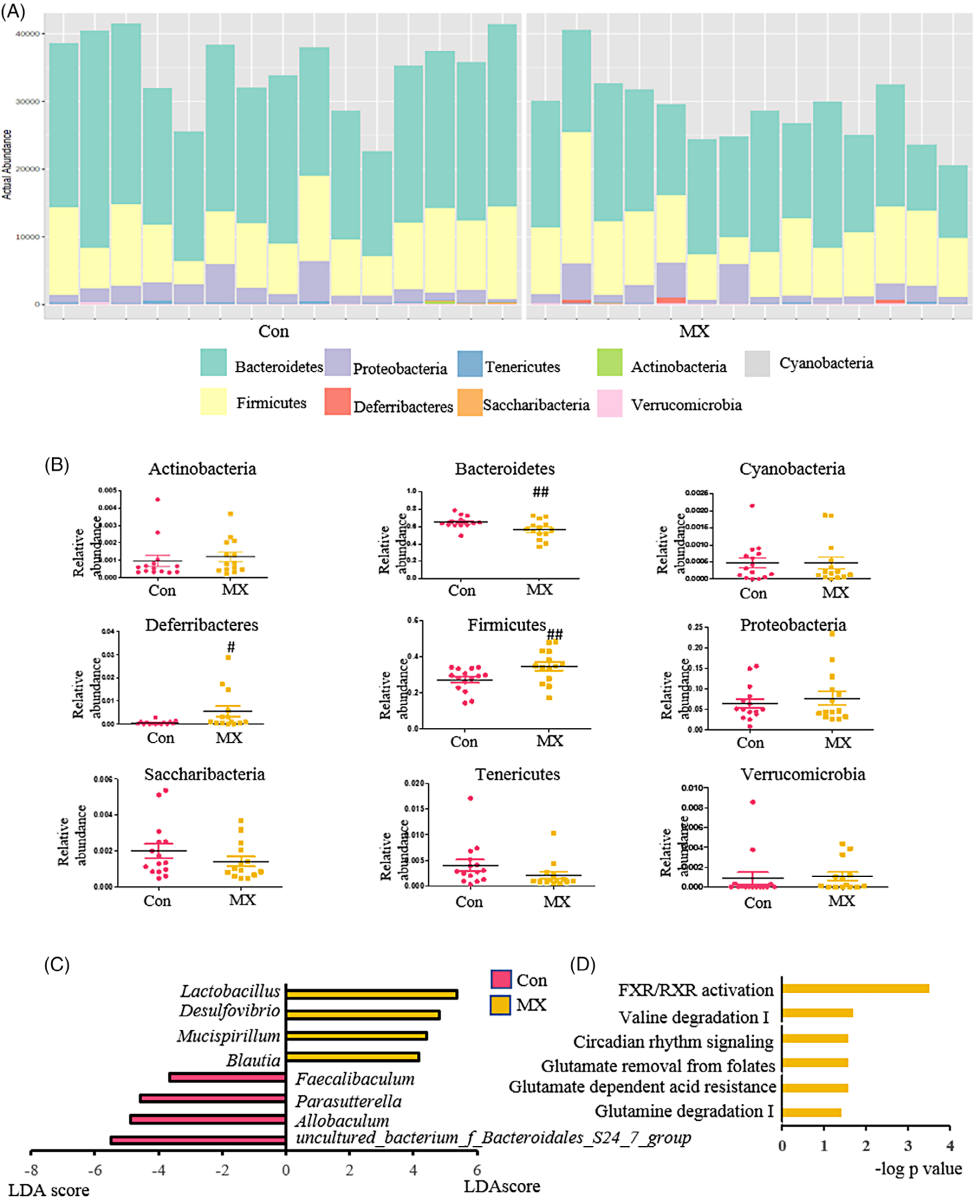
The effect of MX on intestinal flora and metabolism in normal mouse. (A) Area plot showing average percentage of bacterial population among control group (Con) and MX‐treated group (MX, 4 g/kg/d) in Phylum level. (B) The relative abundance of bacteria in Phylum level. (C) Histogram of the LDA scores computed for features differentially abundant. Only taxa meet an LDA significant threshold > 3 and *p* < 0.05 were shown; (D) metabolic pathway enrichment analysis of differential metabolites.

MX is a purgative drug in TCM, and it is essential to be sure what will be brought about by continuous oral administration of MX. Therefore, we investigated the effect of MX on normal healthy mice by administration for 7 days. The changes in microscopic physiological state can give hints about the true effects of MX, and the high‐throughput sequencing technology was used to detect the structure of gut microbes. During the administration, all the sequences were divided into operational taxonomic unit (OTU) based on different levels of similarity, which was used to classify groups of closely related individuals. The results demonstrated that MX affected the diversity of the gut bacteria in normal mice (Figures 6A and B). Through the analysis at phylum level, the microorganisms with the highest LDA score was *Lactobacillus* in MX group (Figure 6C). Moreover, under the optimal LC–MS conditions described above, the typical base peak intensity chromatograms of feces were obtained from all groups (Figures S12 and S13). Orthogonal partial least squares‐discriminant analysis (OPLS‐DA) showed obvious separation between control and MX‐treated groups, which suggested that biochemical disturbance occurred significantly in MX‐administrated group (Figure S14). The results demonstrated that 35 differential metabolites, and BAs metabolism was the critical pathway affected by MX (Figure S15 and Tables S13–S16). Besides, FXR would be the target of MX, even though it was the indirect way (Figure 6D). Generally speaking, healthy mice given continuous gavage of MX did not have pathological changes. Moreover, MX regulated intestinal homeostasis, especially *Lactobacillus*, as well as with significant changes in BA metabolism in the metabolic phenotype.

### 2.6 MX regulated *Lactobacillus*–BA–intestinal FXR axis

**FIGURE 7:**
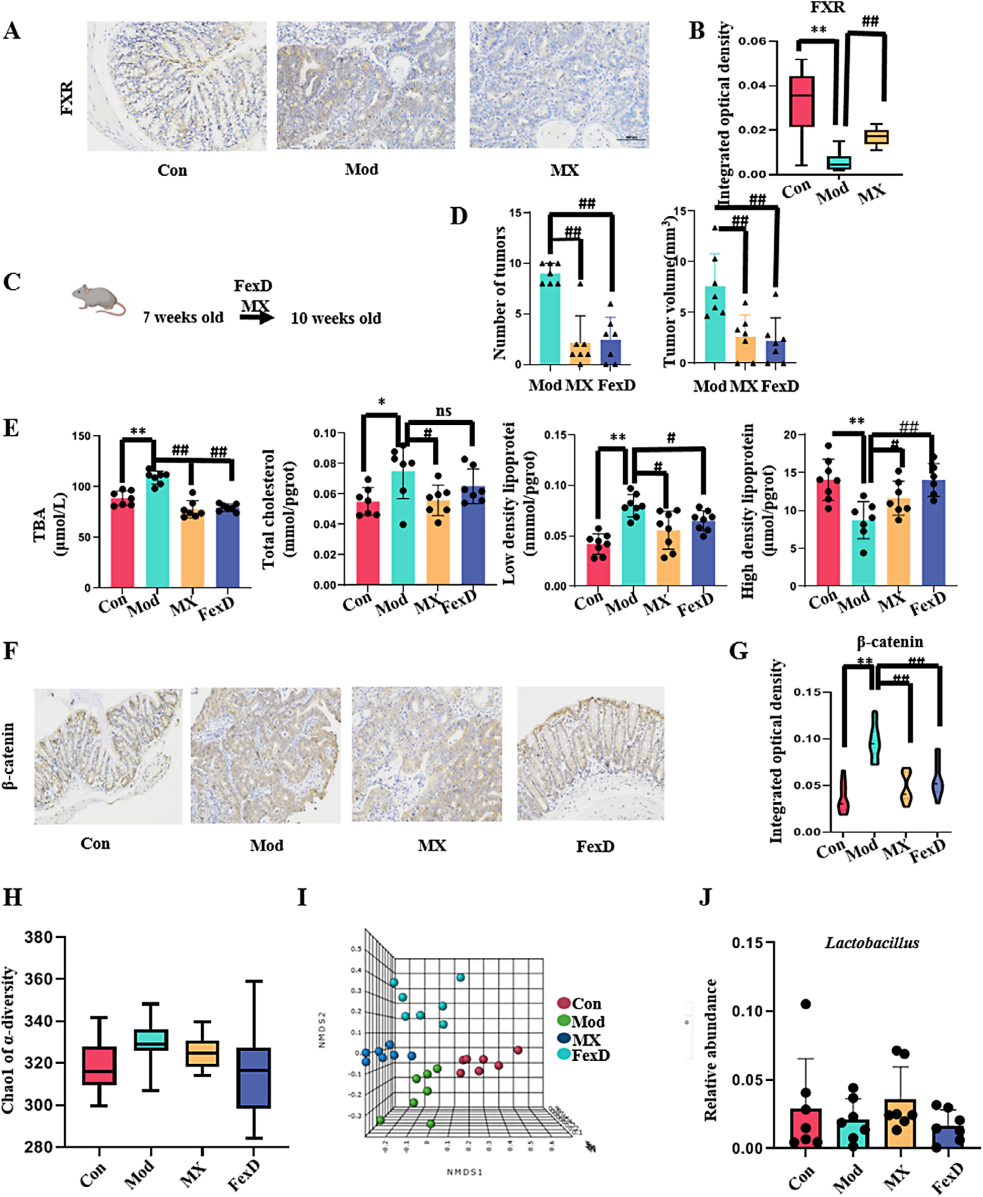
The relationship of the effects of MX and FexD on *Lactobacillus*–FXR axis. (A) Immunohistochemically observation of FXR among control (Con), model (Mod), and MX‐treated group (MX, 4 g/kg/d) in the prevention phase. (B) The expression of FXR. (C) Transgenic mice were given MX (MX, 4 g/kg/d) or FexD (FexD, 50 mg/kg/d) from 7 weeks old age for 21 days. (D) The comparison of the number of tumors, and volume among model group (Mod), MX‐treated group (MX, 4 g/kg/d), and FexD group (FexD, 50 mg/kg/d). (E) The levels of TBA, T‐CHO, LDL‐C, and HDL‐C. (F) and (G) Immunohistochemically observation and the expression of β‐catenin in the prevention phase; (H) comparison of α‐diversity. (I) Comparison of β‐diversity. (J) The comparison of the *Lactobacillus*.

**FIGURE 8:**
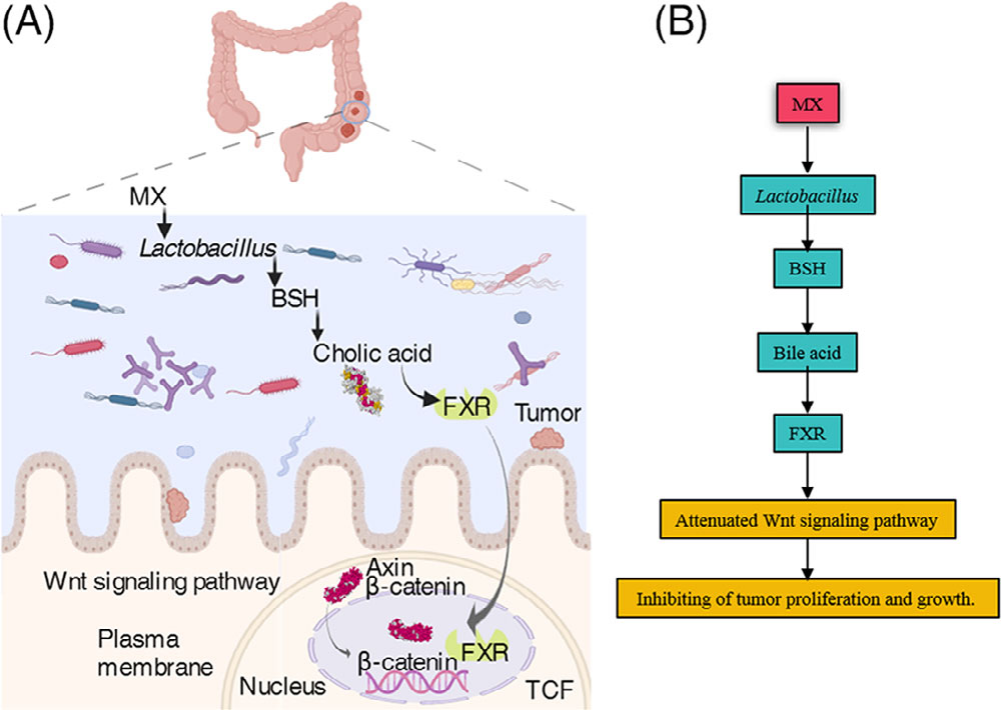
Schematic representation of hypotheses concerning the mechanisms underlying the role of the *Lactobacillus*–bile acid–FXR axis in regulating CRC.

The activation of FXR by MX continued in the whole process of intestinal tumor formation (Figures 7A and B). Furthermore, APC^Min/+^ mice were administered with fexaramine (FexD), an FXR agonist, for 21 days (Figure 7C). The MX group was given parallel administration during the 21‐day experimental period. During the intervention phase, FexD acted antitumor activity similar to MX, which can reduce the number and volume of tumors (Figure 7D). Biochemical indices were used to evaluate BA expression in the liver, and the results showed that FexD significantly reduced the levels of total bile acid (TBA), low‐density lipoprotein cholesterol (LDL‐C), and high‐density lipoprotein cholesterol (HDL‐C; Figure 7E). We also found that FexD could effectively control the expression of β‐catenin (Figures 7F and G). In APC^Min/+^ mice, the supplementation with FexD mimicked the effects of MX on BA metabolism and intestinal β‐catenin expression. By analyzing the gut microbiota α‐diversity and β‐diversity, FexD, like MX, could also callback the abnormal intestinal flora (Figures 7H and I). However, in the further analysis of the key microorganism, *Lactobacillus* was not the main bacteria and FexD had no positive regulation on it (Figure 7J). It showed that the increase of *Lactobacillus* was not caused by the activation of FXR, but the direct effect of MX. Taken together, it is concluded that MX could inhibit the occurrence and development of CRC by regulating the growth of *Lactobacillus* and directly affect the BA–FXR axis (Figures 8A and B).

## 3 DISCUSSION

CRC is one of malignant tumors with high rate of incidence and mortality.^29^, ^30^ The diagnosis and treatment of CRC is currently really nonideal in clinical setting due to the complex pathogenesis, and most cases of the patients are diagnosed at the late stages of CRC. Hence, there is an urgent need for the discovery and development of effective drugs. In recent studies, gut microbiota is evidenced to highly correlate with CRC progression, and it was noticed that osmotic pressure also affects the composition and structure of intestinal flora as well. Therefore, the changing diversity of gut microbiota indeed affects the progression of colon diseases, such as adenoma, polyp, colon cancer, and so on.^31^, ^32^ In this study, we first evaluated the effectiveness of MX on intestinal adenomas and CRC, and multiomics integration analysis revealed that MX can affect the host gut microbiota and its metabolism. We also demonstrated that MX fulfill the role by regulating the *Lactobacillus*–BA–FXR axis. This discovery elucidates the mechanism of action of the difficult to absorb mineral drug MX, which can regulate CRC by affecting gut microbiota and metabolism.

Both clinical and experimental studies have demonstrated MX has the capacity to inhibit the CRC tumors; however, pharmacological mechanism remains incompletely elucidated.^4^, ^12^, ^33^ In our study, we confirmed again that MX can significantly intervene the occurrence and development of CRC characterized by the reduced number and volume of intestinal adenoma, the improved mechanical barrier structure of the intestine and the increased the gland‐to‐velvet ratio (Figures 1 and 5), especially in the middle‐dosage group, which is equivalent to twice the clinical dosage, and in subsequent studies, we adopted the medium dose (4 g/kg/d) as the optimal dose for our study. Basically, inflammatory reactions are involved in different stages of tumor development, and chronic inflammation plays a major role in the occurrence and development of CRC. In addition, the occurrence of CRC is also accompanied by the abnormal expressions of tumor markers including CEA, CA199, and CA242. Our data manifested that MX could regulate the disorder of inflammatory factors and abnormal tumor markers (Figures 1 and 5).

Through gut metabolomics analysis, we found that MX is capable of regulating both host and gut microbiota metabolism, and most of metabolic pathways were linked to intestinal flora (Figure 1). Thus, high‐throughput sequencing technology was used to analyze the effects of MX on intestinal flora. During the treatment of CRC at different stages, MX was verified to restore abnormal intestinal flora and the bacterial strain *Lactobacillus* was mostly affected (Figures 3, 4, 5, 6). Furthermore, similar bacterial phenotype was identified with healthy‐control mice (Figures 6A–C). Sugimura et al.^34^ discovered *L. gallinarum* protected against intestinal tumourigenesis by producing protective metabolites that could promote CRC cells apoptosis. Chung et al.^35^ also found Lactic acid bacteria were ideal candidates for live vehicles engineered to deliver anticancer drugs.

BA metabolism was widely influenced by gut microbiota.^36^, ^37^ Wang et al.^38^ found the imbalance of gut microbiota‐induced abnormalities in BA can promote the intestinal adenoma‐adenocarcinoma. Besides, it was also the key pathway for intermediating antitumor effects of anti‐CRC drugs.^39^ We found that MX intervening the progression of CRC by mainly targeting BA metabolism. Gut‐microbiota 16S rDNA sequencing analysis confirmed that MX exerts therapeutic effects by changing the abundance of *Lactobacillus* and upregulating BSH to increase the expressional levels of unconjugated BAs in APC^Min/+^ mice (Figures 4B–D and 5I and J). Chenodeoxycholic acid and its derivatives have been approved by the US FDA as new drugs that activate FXR and inhibit the dysregulated bile metabolism.^40^ The present study found that chenodeoxycholic acid was increased by BSH after the treatment of MX (Figure 5J), indicated MX exerts the therapeutic effect by affecting the BSH regulated by *Lactobacillus*, and then regulates BA metabolism.

Through the enrichment analysis of key metabolic pathways, it was found that MX could affect FXR receptors regardless of the stage of treatment (Figures 4C and S10C). FXR has multiple biological functions, and it is highly expressed in the liver and intestines. Multiple studies demonstrated that intestinal FXR could affect the host's function by the regulation of various metabolic pathways. Fu et al.^41^ discovered that FXR was a potential therapeutic target for CRC. During the intervention phase of MX, the expression of FXR was indeed activated by the influence of MX (Figures 7A and B). To verify whether FXR could reverse the occurrence of CRC, we used FexD, the agonist of FXR, to inhibit intestinal adenoma in APC^Min/+^ mice, and found that the number and volume of intestinal adenomas were significantly reduced (Figure 7C). It can be seen that the activation of FXR can indeed play a role in inhibiting the occurrence of intestinal adenomas. In addition, the increase of BAs of intestinal adenoma stage such as TBA, T‐CHO, LDL‐C, and HDL‐C was found to be inhibited by FexD that is agreeable with the anti‐CRC effect of MX (Figure 7E).

The model mice used in present study originates from the APC mutation, which activates β‐catenin and ultimately promotes the progression of intestinal tumors, and β‐catenin is the key protein of the Wnt signal pathway.^28^ Furthermore, MX, like FexD, could inhibit the key protein β‐catenin (Figures 7F and G). A recent study found that the metabolites of BAs produced by microorganisms could attenuate the DNA‐binding activity of β‐catenin/TCF4 by activating FXR, and subsequently inhibit tumor proliferation and growth.^42^ In order to further verify whether the direct target of MX is gut microbiota or FXR, high‐throughput sequencing technology was employed to clarify the relationship among MX, *Lactobacillus*, and FXR. The results revealed that FexD did not promote abundance of *Lactobacillus* (Figure 7J), while MX did, indicating the direct target of MX was *Lactobacillus*. The results of genetic abundance of *Lactobacillus* also confirmed that MX affected the BA‐related biological network through changing the abundance of *Lactobacillus* (Figure 5J). The overall data concluded that MX as a mineral drug had a similar probiotic agonist effect and could attenuate CRC pathological progression by regulating *Lactobacillus*–BA–intestinal FXR axis.

In summary, we were the first to evaluate the effectiveness of MX on intestinal adenomas and CRC, further multiomics integration analysis revealed that MX can restore the dysregulated gut microbiota and associated metabolism in CRC mice, in which MX exert the role of anti‐CRC by mostly regulating the *Lactobacillus*–BA–FXR axis. Such discovery might help to decipher the pharmacological mechanism of MX with a lower bioavailability, but it can exert significantly inhibitory role on CRC tumor by targeting the dysregulate gut microbiota and associated metabolism. Moreover, the limitation of this experiment lied in the absence of data on the expression of the microenvironment in the human intestinal tract after the administration of MX in clinical settings. We were looking forward to being updated experimental data in the future to support this conclusion.

## 4 CONCLUSION

Our investigation had provided partial elucidation on the inhibitory mechanisms employed by MX in preventing the onset and progression of CRC through spontaneous intestinal adenoma. Our findings supported the hypothesis that MX exerted its inhibitory effects on CRC by modulating the gut microbiota, specifically via the *Lactobacilli*–BA–intestinal FXR axis. Notably, MX exhibited a probiotic agonist effect. Furthermore, our identification of FXR as the key target of MX within the gut underscores its potential as a promising therapeutic target for CRC treatment. This study contributes valuable insights into the intricate pathways through which MX might mitigate CRC, paving the way for potential therapeutic interventions targeting the gut microbiota and the FXR receptor.

## 5 MATERIALS AND REAGENTS

MX (purity > 99%) was provided by TongRentang Pharmacy (Harbin, China) and identified by Prof. Wang Xijun, and the voucher specimen is deposited in Heilongjian University of Traditional Chinese Medicine. FexD was provided by Medchem Express (Monmouth Junction, NJ, USA). Acetonitrile and methanol were obtained from Thermo Fisher Scientific (Waltham, MA, USA), and ultrapure water was produced by Milli‐Q ultra‐pure water system (Merck Millipore, Burlington, MA, USA). The ELISA kits of IL‐6, TNF‐α, IFN‐γ, CEA, CA199, and CA242 were purchased from Thermo Fisher Scientific. Beta‐catenin kits were provided by Thermo Fisher Scientific.

### 5.1 Animal handling

Six‐week‐old APC^Min/+^ male mice (18–20 g) were supplied by the Nanjing Biomedical Research Institute of Nanjing University (Nanjing, China). Mice were housed under controlled room temperature (25 ± 1°C), humidity (50 ± 5%), and light (12/12 h light/dark cycle) and received chow and tap water ad libitum. APC^Min/+^ male mice were divided into four groups at 7 weeks of age: (1) model group (Mod, *n* = 20), mice received chow and tap water ad libitum; (2) MX low dose group (MX‐L, *n* = 20), mice daily administration of MX at low dose of 2 g/kg/d; (3) MX middle dose group (MX‐M, *n* = 20), mice daily administration of MX at middle dose of 4 g/kg/d; (4) MX high dose group (MX‐H, *n* = 20), mice daily administration of MX at high dose of 6 g/kg/d. The preparation of the MX is described in the supplementary material, and MX was gavaged with 0.01 mL/g body weight every day for 3 weeks. For the comparison, 7‐week‐old C57BL/6J mice, which were fed with chow and tap water ad libitum, were used as control group (Con, *n* = 20). All of mice were sacrificed at 10 weeks of age, and samples were collected. The number, size, diameter, and weight of tumors were determined. The tumors were collected and fixed in 10% buffered formalin for subsequent analysis.

In the next experiment, APC^Min/+^ male mice were divided into two groups at 10 weeks of age: (1) model group (Mod, *n* = 20), mice received chow and tap water ad libitum; (2) MX group (MX, *n* = 20), mice daily administration of MX at dose of 4 g/kg/d. For the comparison, 10‐week‐old C57BL/6J mice, which were fed with chow and tap water ad libitum, was used as control group (Con, *n* = 20). All of mice were sacrificed at 20 weeks of age and samples were collected. The number, size, diameter, and weight of tumors were determined. The tumors were collected and fixed in 10% buffered formalin for subsequent analysis.

ICR male mice (20 ± 2 g) were supplied by the GLP Center of Heilongjiang University of Chinese Medicine (Harbin, China). They had free access to food pellets and tap water in controlled room. All animals were allowed to acclimatize in metabolism cages for 1 week prior to the treatment. All mice were randomly divided into two groups, including control group (Con, *n* = 15) and MX group (*n* = 15). The dosage of MX was 4 g/kg/d. The administration group was administrated every day for 7 days.

For FXR agonist validation experiments, 6‐week‐old APC^Min/+^ male mice (18–20 g) were supplied by the GemPharmatech LLC. (Nanjing, China). Mice were divided into three groups at 7 weeks of age: (1) model group (Mod, *n* = 7), mice received chow and tap water ad libitum; (2) FexD group (FexD, *n* = 7), mice daily administration of FexD at dose of 50 mg/kg/d; (3) MX group (MX, *n* = 7), mice daily administration of MX at dose of 4 g/kg/d. The preparation of the FexD is described in the supplementary material, and MX or FexD oil solution was gavaged with 0.01 mL/g body weight every day for 3 weeks. For the comparison, 7‐week‐old C57BL/6J mice, which were fed with chow and tap water ad libitum, was used as control group (Con, *n* = 7). All of mice were sacrificed at 10 weeks of age and samples were collected. The number, size, diameter, and weight of tumors were determined. The tumors were collected and fixed in 10% buffered formalin for subsequent analysis. We purchased three batches of MX, and the content of main component sodium sulfate was all greater than 99.0%. The specific measurement method and content were provided in the supplementary data (Table S17).

### 5.2 Sample preparation

In different stages of our experimental design, all the fecal samples was using the same way. Feces were collected at 8:00 am, which were then transferred into a set of clean tubes and stored in liquid nitrogen for metabolomics study.

In the intervention experiment of MX, treatment experiment of MX, and the confirmatory experiment of FXR, blood was collected after the final administration by pricking from the right eyeball delivered into Eppendorf tubes. After standing for 30 min at 4°C, the blood samples were centrifuged at 825*g* , 4°C for 20 min and detached serum section was collected and stored frozen at −80°C until the next analysis step.

In the intervention and treatment experiment of MX, the small intestine, cecum, and colorectal were resected via an abdominal incision. Intestinal content, cecum content, and colorectal content were obtained and stored at −80°C until the next analysis step. An intestine contents mixed sample was prepared as the quality control sample that was used in the optimization of the metabolomics analysis method. The small intestine and colon were harvested, flushed immediately with ice‐cold physiological saline, and longitudinal split. Intestinal tumors were examined under a dissection microscope.

The fixed intestine tumor samples were dehydrated in a grade alcohol series, embedded in paraffin wax, and sectioned at 5 μm. Tissue was stained with standard H&E for the light microscopic examination.

In the FXR agonist validation experiments, the mouse liver was extracted. The samples were washed in normal saline, immediately frozen in liquid nitrogen, and then were stored in a refrigerator at −80°C. During testing and analysis, weigh 0.2 g of liver tissue and add 1.8 mL of saline.

### 5.3 Preparation of metabolomics samples

Before serum metabolomics analysis, the serum samples were thawed at room temperature. One hundred microliters thawed serum was mixed with 100 μL methanol, vortexed for 1 min, and then centrifuged at 15,493*g* at 4°C for 10 min for the supernatant acquisition. All feces specimens were collected into sample containers and stored at −80°C until the analysis. During the extraction of fecal, weighted samples were thawed at room temperature and were mixed with extraction buffer (50% acetonitrile and 50% water) at a ratio of 100 mg/mL. The mixtures were vigorously swirled for 60 s and centrifuged at 15,493*g* for 10 min. All small intestine content, cecum content, and colorectal content were separated collected into samples containers and stored at −80°C until the analysis. During the extraction of small intestine content, cecum content, colorectal content, and weighted samples were thawed at room temperature and were separated and mixed with extraction buffer (50% acetonitrile and 50% water) at a ratio of 10 mg/mL. The mixtures were vigorously swirled for 60 s and centrifuged at 15,493*g* at 4°C for 10 min. The supernatants of all samples were filtered through a membrane (0.22 μm) and were injected directly into the column for UPLC–QTOF–MS analysis.

### 5.4 UPLC analysis

In serum metabolomics, the chromatographic separation was performed on an Acquity UPLCTM system (Waters, Milford, USA). An ACQUITY UPLC BEH C18 column (100 mm × 2.1 mm i.d., 1.8 μm; Waters) was used. The column was set at 40°C with the flow rate of 0.3 mL/min. The sample injection volume was 2 μL. The optimal mobile phase consisted of a linear gradient system of (A) 0.1% formic acid in acetonitrile and (B) 0.1% formic acid in water: 0–1.5 min, 2–16% A; 1.5–2 min, 16–20% A; 2–4 min, 20–60% A; 4 −4.5 min, 60–65% A; 4.5–8 min, 65–70% A; 8–10 min, 70–100% A; 10–12 min, 100% A; 12–14 min, 100–2% A. The sample storage room temperature was 4°C.

In the metabolomics of fecal sample and intestinal contents, the chromatographic separation was performed using the same system except for the flow rate of 0.4 mL/min and the linear gradient system as follows: (A) 0.1% formic acid in acetonitrile and (B) 0.1% formic acid in water: 0–1 min, 1–50% A; 1–2.5 min, 50–90% A; 2.5–6.5 min, 90–100% A; 6.5–8.0 min, 100% A; 8.0−10.5 min, 100–1% A; 10.5–11.0 min, 1% A.

### 5.5 MS analysis

In serum metabolomics, the eluent was introduced into the high‐definition mass spectrometer (Waters SynaptTM G2‐Si MS; Waters) analysis, and the optimal conditions of analysis were as follow: in electrospray ionization (ESI)^+^ mode, the capillary voltage was 3.0 kV, the sampling cone voltage was 20 V, desolvation temperature was 350°C, desolvation gas flow was 800 L/h; in ESI^–^ mode, the capillary voltage was 3.0 kV, the sampling cone voltage was 20 V, desolvation temperature was 350°C, desolvation gas flow was 1000 L/h. The data acquisition rate was fixed to 0.4 s/scan with 0.1 s inter scan delay. The mass range was set at *m*/*z* 50–1500 using extended dynamic range. Leucine‐enkephalin was used for ensuring accuracy during the whole experiment.

In the metabolomics of fecal sample and intestinal contents, the eluent was introduced into the same condition except for the sampling cone voltage of 30 V and desolvation gas flow of 600 L/h for ESI^+^; and for the capillary voltage was 2.6 kV and desolvation gas flow of 600 L/h for ESI^–^.

### 5.6 Metabolite identification and pathway analysis

By applying the Micromass MarkerLynx version 4.0 (Waters), the LC–MS raw data were managed and the data were analyzed by Progenesis® QI software (Waters). The PCA and PLS‐DA were achieved to evaluate the MS data by generating the cluster of groups. PCA and PLS‐DA were performed using the Waters EZinfo software. The following analysis with OPLS‐DA scores was conducted to analyzing the relative intensity of metabolites, and VIP‐plot was used to identify the candidate metabolites. MassFragment™ application manager (Waters) was applied to characterize the MS/MS fragments of metabolites. The combination approaches of Metlin, HMDB, and ChemSpider were conducted to analyze the biomarkers and revealing the related biological information.

### 5.7 High‐throughput sequencing and functional analysis

The DNA of the microbiota was extracted from 200 mg fecal sample by using a PowerSoil® DNA Isolation Kit (Omega, Shanghai, China). A total amount of 1 μg DNA per sample was used as input material for the DNA sample preparations. Sequencing libraries were generated using NEBNext® Ultra™ DNA Library Prep Kit for Illumina (New England BioLabs, Ipswich, MA, USA) following manufacturer's recommendations and index codes were added to attribute sequences to each sample. Briefly, the DNA sample was fragmented by sonication to a size of 300 base pair, then DNA fragments were end‐polished, A‐tailed, and ligated with the full‐length adaptor for Illumina sequencing with further polymerase chain reaction (PCR) amplification. At last, PCR products were purified (AMPure XP system, Beckman Coulter, Brea, CA, USA) and libraries were analyzed for size distribution by Agilent 2100 Bioanalyzer (Agilent, Santa Clara, CA, USA) and quantified using real‐time PCR. The amplicon library preparation was performed by PCR amplification of the V3–V4 region of the 16S rDNA gene. The universal primers 338F (5′‐ACTCCTACGGGAGGCAGCAG‐3′) and 806R (5′‐GGACTACHVGGG TWTCTAAT‐3′) were used. PCR reactions were conducted in a thermocycler PCR system (PCR Sprint; Rockwell Automation, Houston, USA) using the following program: 5 min of denaturation at 95°C, followed by 15 cycles of 95°C for 1 min, 50°C for 1 min, and 72°C for 1 min, along with an extension at 72°C, for 7 min and storage at 4°C. The clustering of the index‐coded samples was performed on a cBot Cluster Generation System NEBNext® Ultra™ II DNA Library Prep Kit (New England BioLabs) according to the manufacturer's instructions. After cluster generation, the library preparations were sequenced on an Illumina HiSeq platform (Illumina, San Diego, CA, USA) at Biomarker Technologies (Rohnert Park, CA, USA). High‐quality reads for bioinformatics analysis were performed and all of the effective reads from each sample were clustered into OTUs based on a 97% sequence similarity according to UCLUST. For diversity analysis, the α‐diversity was performed using Mothursoftware (version v.1.30). The relative abundance of bacteria was used Microbiomeanalyst network.

### 5.8 Metagenomics analyses and functional analysis

The DNA extraction method and library construction were shown in the previous above. We obtained a total of 440 Gb of raw paired‐end reads. The taxonomic and KO composition was obtained by using an updated version of the MEDUSA pipeline, in which the raw reads were trimmed by FASTQ, filtered to remove human reads, and then mapped to bacterial gene and genome catalogs. An additional filter was applied during the mapping process containing reads with at least 85% identity to obtain high‐quality reads. The mean mapping rates for the genome and gene catalogs were 20 and 83%, respectively. BLASTP software was used to align the gene sequences of nonredundant gene set with the NR database and KEGG to obtain annotation information.

### 5.9 Statistical analysis

The heatmaps were generated to visualize the intestinal microbiome differences among each group. The Benjamin Hochberg method was used for FDR control (FDR < 0.05). The comparison between two groups were tested by two‐tailed Student's *t*‐test. The comparison among multiple groups were tested by one‐way analysis of variance followed by Dunett's *t*‐test. Significant expression profiles were identified with a false discovery rate of 0.05. Data were expressed as the mean ± standard deviation as indicated in the figure legends. Statistical analysis was conducted using IBM SPSS Statistics 24 software and Excel. The figures were created using GraphPad Prism 8 software.

## AUTHOR CONTRIBUTIONS

Xijun Wang conceived and designed the experiments. Honglian Zhang, Xinghua Li, Fengting Yin, Jing Li, and Xiaohang Zhou mainly conducted this study. Xiaohang Zhou, Le Yang, and Honglian Zhang provided help on gut microbiota analysis. Xinghua Li, Fengting Yin, and Jing Li provided help on metabolomics analysis. Xiaohang Zhou and Hui Sun drafted the manuscript. Xijun Wang, Junling Ren, Haitao Lu, Guangli Yan, and Toshiaki Makino provided manuscript revision. All authors reviewed and approved the final manuscript.

## CONFLICT OF INTEREST STATEMENT

All authors have read the journal's authorship agreement and policy on the disclosure of potential conflict of interests. There was no conflict of interest.

## ETHICS STATEMENT

All animals were conducted in compliance with the Ethical Committee of Heilongjiang University of Chinese Medicine (approval number: HUCM‐2016028).

## Supporting information

Supporting Information

## Data Availability

All raw sequencing data from this study can be accessed at the National Center for Biotechnology Information (NCBI) under the bioproject of ID PRJNA1087540.
