# Supplementary material for: Mineral crude drug mirabilite (Mangxiao) inhibits the occurrence of colorectal cancer by regulating the Lactobacillus–bile acid–intestinal farnesoid X receptor axis based on multiomics integration analysis
Source: MedComm (2020). 2024 Apr 25;5(5):e556. doi: 10.1002/mco2.556 (PMC11043829; doi:10.1002/mco2.556)
Supplement: Supplementary file 1 — Supporting Information [file MCO2-5-e556-s001.docx]

**Supplementary material for Mineral crude drug mirabilite (Mangxiao) inhibits the occurrence of colorectal cancer by regulating the Lactobacillus-bile acid-intestinal farnesoid X receptor axis based on multi-omics integration analysis**

Xiaohang Zhou^1^, Hui Sun^1^, Junling Ren^1^, Guangli Yan^1^, Le Yang^2^, Honglian Zhang^3^, Haitao Lu^4,5^, Xinghua Li^1^, Toshiaki Makino^6^*, Fengting Yin^1^, Jing Li^1^, XijunWang^1,2 *^

^1^ State Key Laboratory of Integration and Innovation of Classical Formula and Modern Chinese Medicine, National Chinmedomics Research Center, National TCM Key Laboratory of Serum Pharmacochemistry, Metabolomics Laboratory, Department of Pharmaceutical Analysis, Heilongjiang University of Chinese Medicine, Heping Road 24, Harbin 150040, China.

^2^ State Key Laboratory of Dampness Syndrome, The Second Affiliated Hospital Guangzhou University of Chinese Medicine, Dade Road 111, Guangzhou, 510260, China

^3^ Pharmacy college, Qiqihar Medical University, No. 333, Kuibei Street, Jianhua District, Qiqihar 161006, China

^4^ School of Chinese Medicine, Hong Kong Baptist University, Hong Kong, China.

^5^ State Key Laboratory of Environmental and Biological Analysis, Hong Kong Baptist University, Hong Kong, China.

6 Department of Pharmacognosy, Graduate School of Pharmaceutical Sciences, Nagoya City University, 3-1 Tanabe-Dori, Mizuho-ku, Nagoya 4678603, Japan.

Email: xijunw@sina.com

**The preparation of medicines**

High-dose group of mangxiao (MX) administration solution was prepared as follows : Powdered MX (0.6 g) was suspended into 1 ml of distilled water solution, and kept 50°C water bath for 10 min.

Middle-dose group of mangxiao administration solution was prepared as follows : Powdered MX (0.4 g) was added into 1ml of distilled water solution, and kept 50°C water bath for 10 min.

Low-dose group of mangxiao administration solution was prepared as follows : Powdered MX (0.2 g) was added into 1ml of distilled water solution, and kept 50°C water bath for 10 min.

Fexaramine (FexD) was prepared as follows: FexD was resolved in corn oil at the concentration of 50 mg/kg.

**Determination of sodium sulfate content in MX**

Dried MX of 1.2 g was add into 20 mL of water, and put the rapid qualitative filter paper into the volumetric flask and dilute it to the scale. Accurately pipetted 25 mL of the above solution and added 75 mL of water and 2 mL of dilute hydrochloric acid, and boiled. Then hot barium chloride was added until no precipitation occurred, and heated on a water bath for 30 min. Filtered with ashless filter paper, and the precipitate was washed with water until the washing solution no longer showed chloride reaction. Dried and ignited the precipitate to constant weight, and calculated the weight of sodium sulfate. The sodium sulfate content in MX was listed in Table S17.

Figure S1 Overview of the workflow integrating multi-biosample metabolomics analysis, intestinal microbial data and mouse feeding experiments

Figure S2 Fingerprint patterns of the intestinal (A and B) and cecal (C and D) contents in control (CON), model (MOD), and MX-treated groups in positive (A and C) and negative (B and D) ion mode.

Figure S3 Fingerprint patterns of the colorectal contents (A and B) and feces (C and D) in control (CON), model (MOD), and MX-treated groups in positive (A and C) and negative (B and D) ion mode.

Figure S4 PLS-DA score plots of fecal samples collected from MX-treated group on different time points. (A) ESI^+^ mode - 2D; (B). ESI^-^ mode - 2D.

Figure S5 PCA score plots for metabolomics analysis of MX regulating host-microbiota co-metabolism in CRC development. Intestinal contents in positive (A) and negative (B) ion mode; cecal contents in positive (C) and negative (D) ion mode; colorectal contents in positive (E) and negative (F) ion mode; fecal contents in positive (G) and negative (H) ion mode. Control group (■), model group (■), MX-treated group (■).

Figure S6 VIP-plots for metabolomics analysis of MX regulating host-microbiota co-metabolism in CRC development. Intestinal contents in positive (A) and negative (B) ion mode; cecal contents in positive (C) and negative (D) ion mode; colorectal contents in positive (E) and negative (F) ion mode; fecal contents in positive (G) and negative (H) ion mode.

Figure S7 The relative intensity among control group(■), model group(■), and MX group (■) in intestinal contents. * *P* < 0.05, ** *P* < 0.01 *vs* control group, ^#^ *P* < 0.05, ^##^ *P* < 0.01 *vs* model group.

Figure S8 The relative intensity among control group(■), model group(■), and MX group (■) in cecal contents. ** *P* < 0.01 *vs* control group, ^#^ *P* < 0.05, ^##^ *P* < 0.01 *vs* model group.

Figure S9 The relative intensity among the control group (■), model group (■) and MX group (■) in colorectal contents. * *P* < 0.05, ** *P* < 0.01 *vs* control group, ^#^ *P* < 0.05, ^##^ *P* < 0.01 *vs* model group.

Figure S10 The relative intensity among the control group (■), model group (■) and MX group (■) in fecal contents. * *P* < 0.05, ** *P* < 0.01 *vs* control group, ^#^ *P* < 0.05, ## *P* < 0.01 *vs* model group.

Figure S11 (A) Heatmap analysis of targeted metabolomics of bile acid metabolism in small intestine contents, cecum contents, colorectal contents and fecal samples; (B) Metabolic pathway enrichment analysis of metabolites; (C) Disease correlation analysis of metabolites.

Figure S12 Fingerprint pattern of the fecal sample of control (A) and MX-treated (B) groups in positive mode.

Figure S13 Fingerprint pattern of the fecal sample of control (C) and MX-treated (D) groups in negative mode.

Figure S14 OPLS-DA scores plot of fecal samples collected from control (■) and MX-treated (■) groups in positive ESI mode (A) and negative ESI mode (B).

Figure S15 The relative intensity among the control (■) and MX group (■) in fecal sample.

Figure S16 Intestinal tumors growth situation in the Mod Group (A), MX-H Group (B), MX-M Group (C), and MX-L (D) Group during the intervention experiment. Tumors growth locations were indicated with black arrows

Figure S17 Intestinal tumors growth situation in the Mod Group (A) and MX (B) Group during the treatment experiment. Tumors growth locations were indicated with black arrows.

Figure S18 Intestinal tumors growth situation in the Mod Group (A), MX (B) Group and FexD (C) during the FexD validation experiment. Tumors growth locations were indicated with black arrows.

**
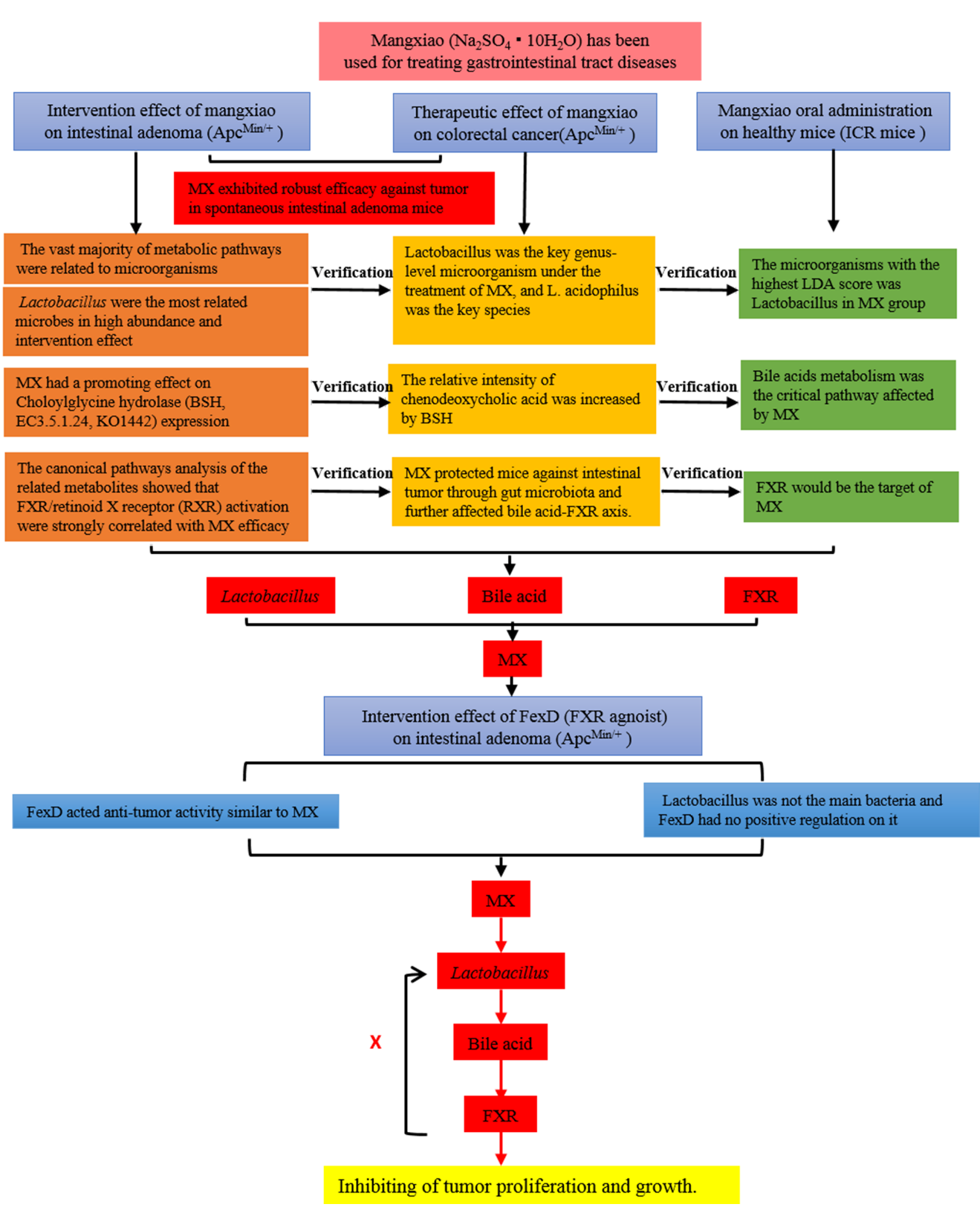
**

**Figure S1**


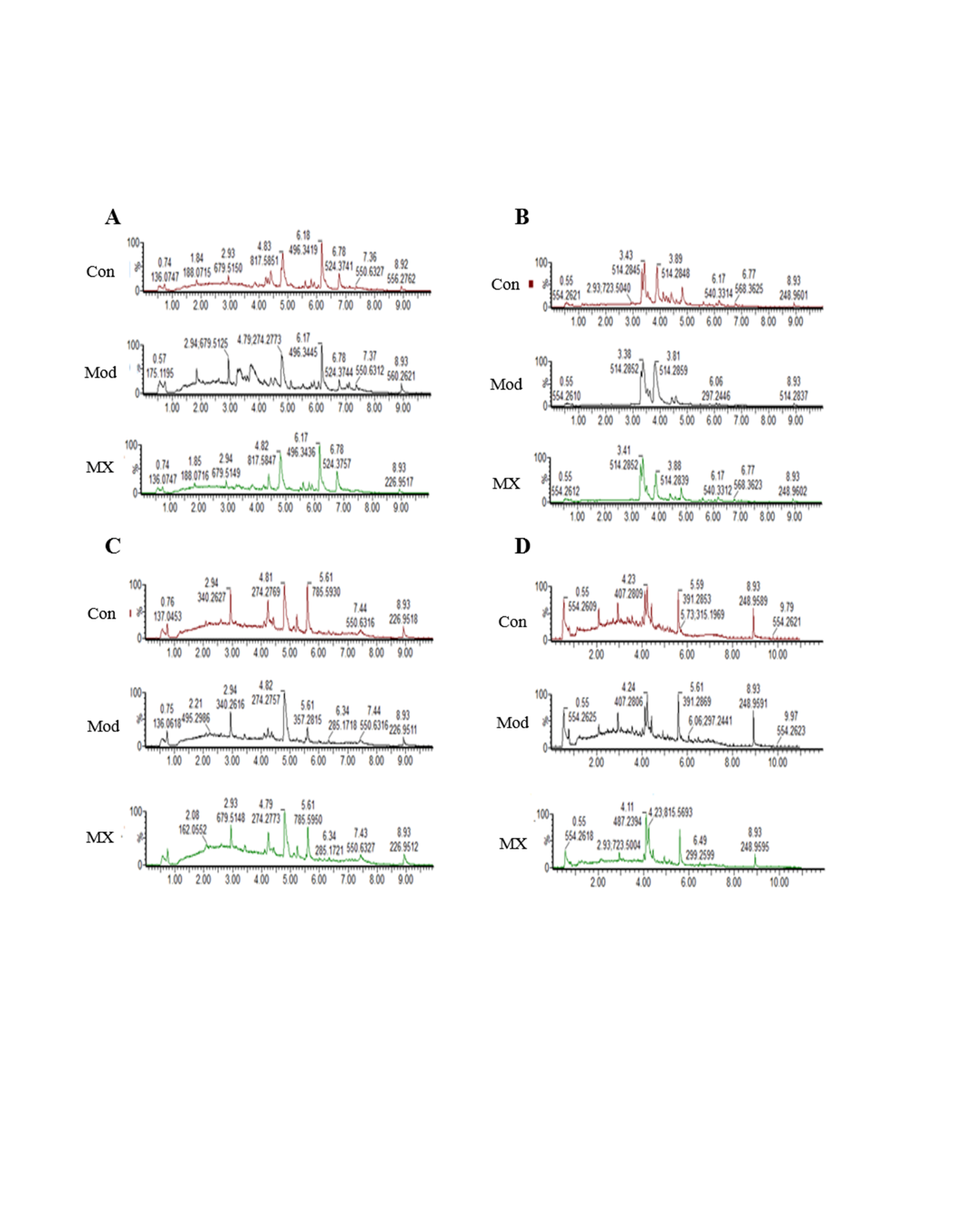


**Figure S2**


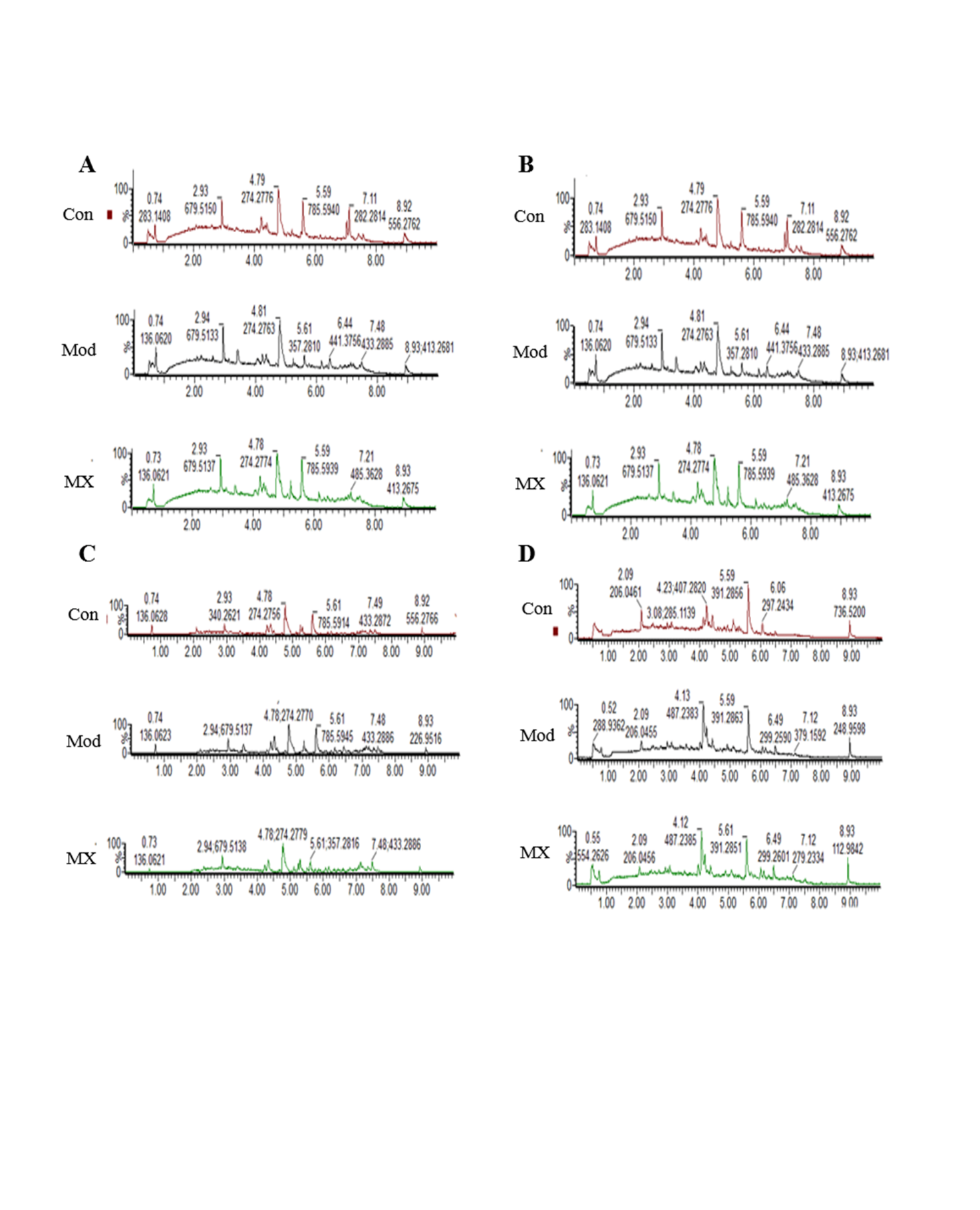


**Figure S3**


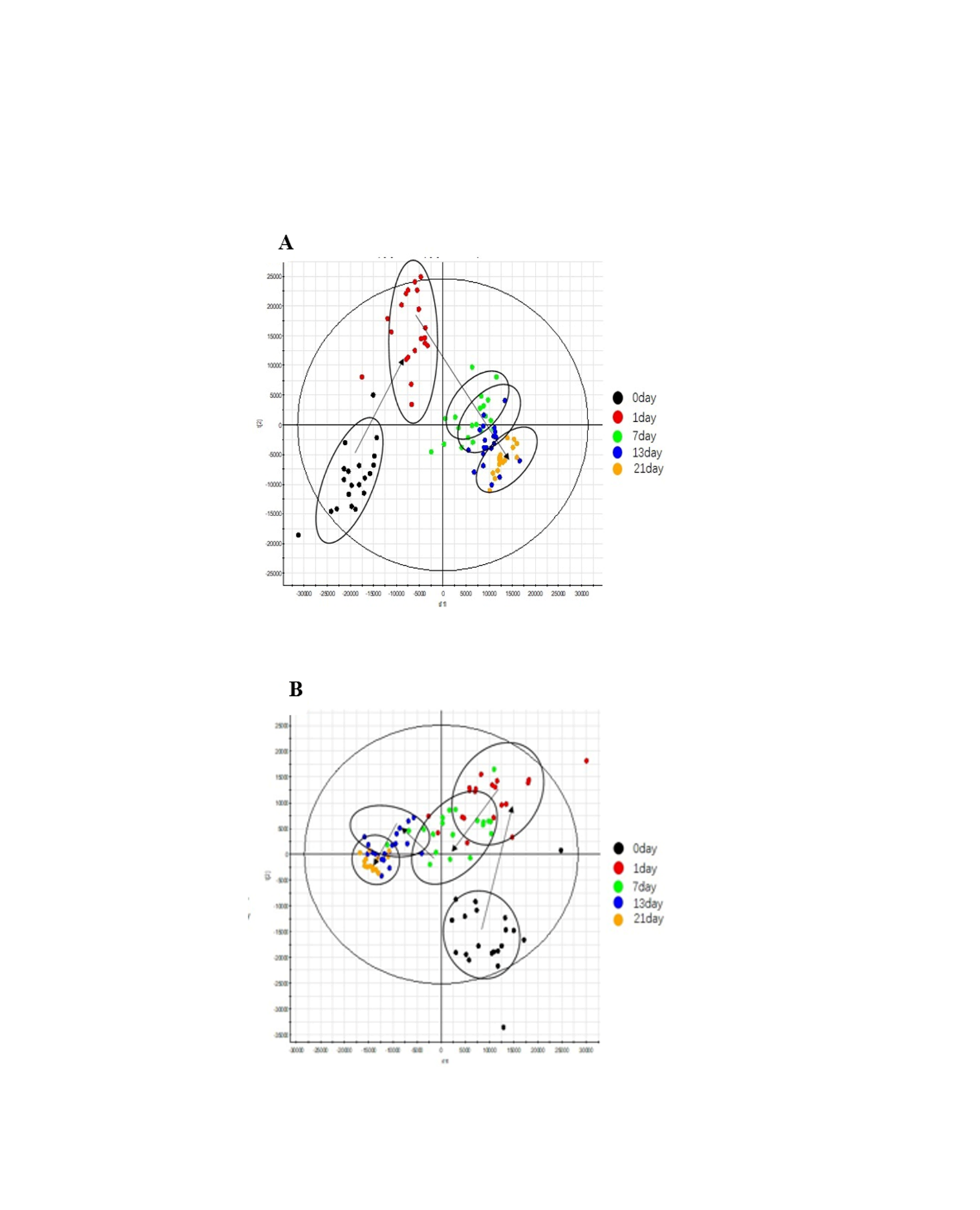


**Figure S4**


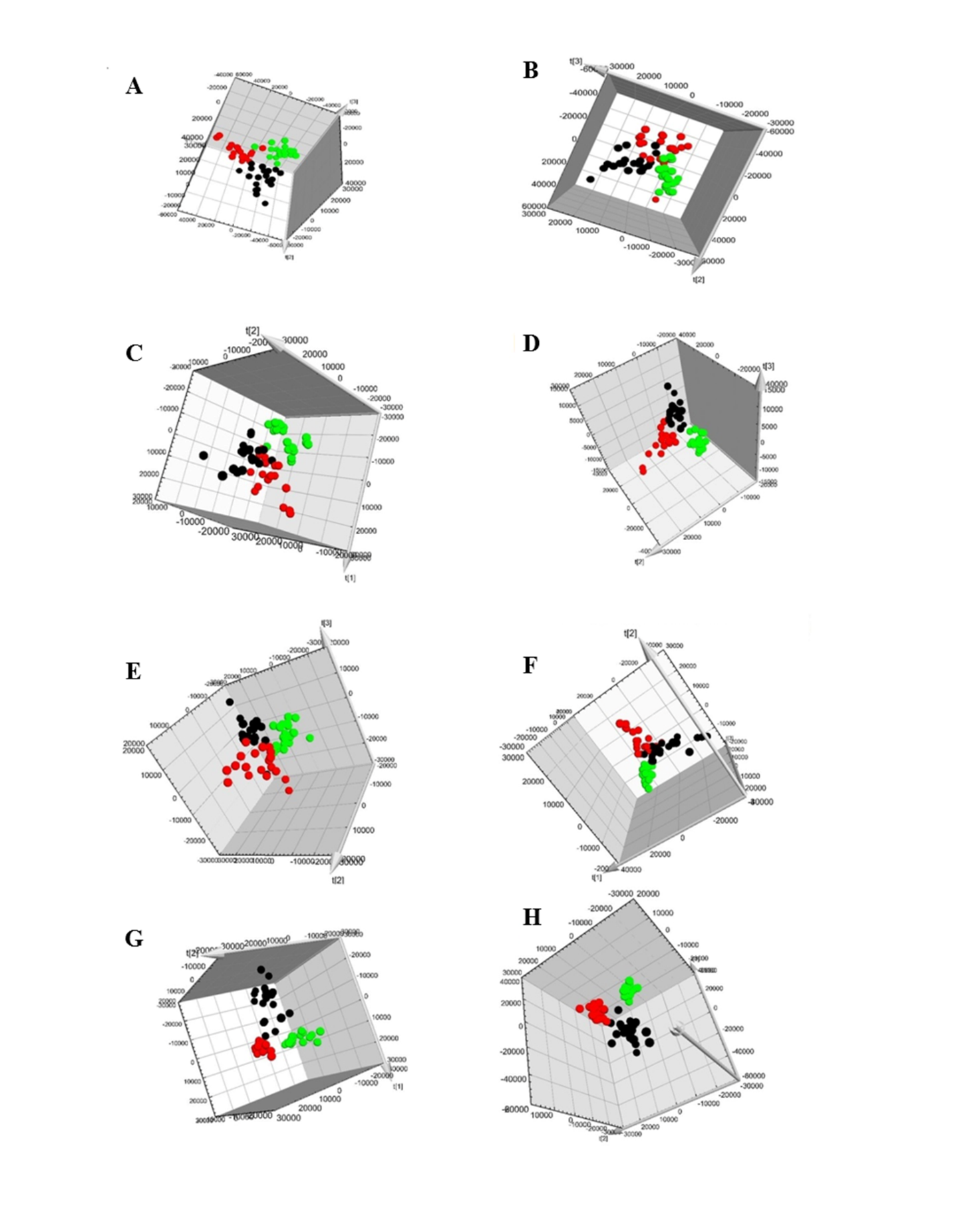


**Figure S5**

**
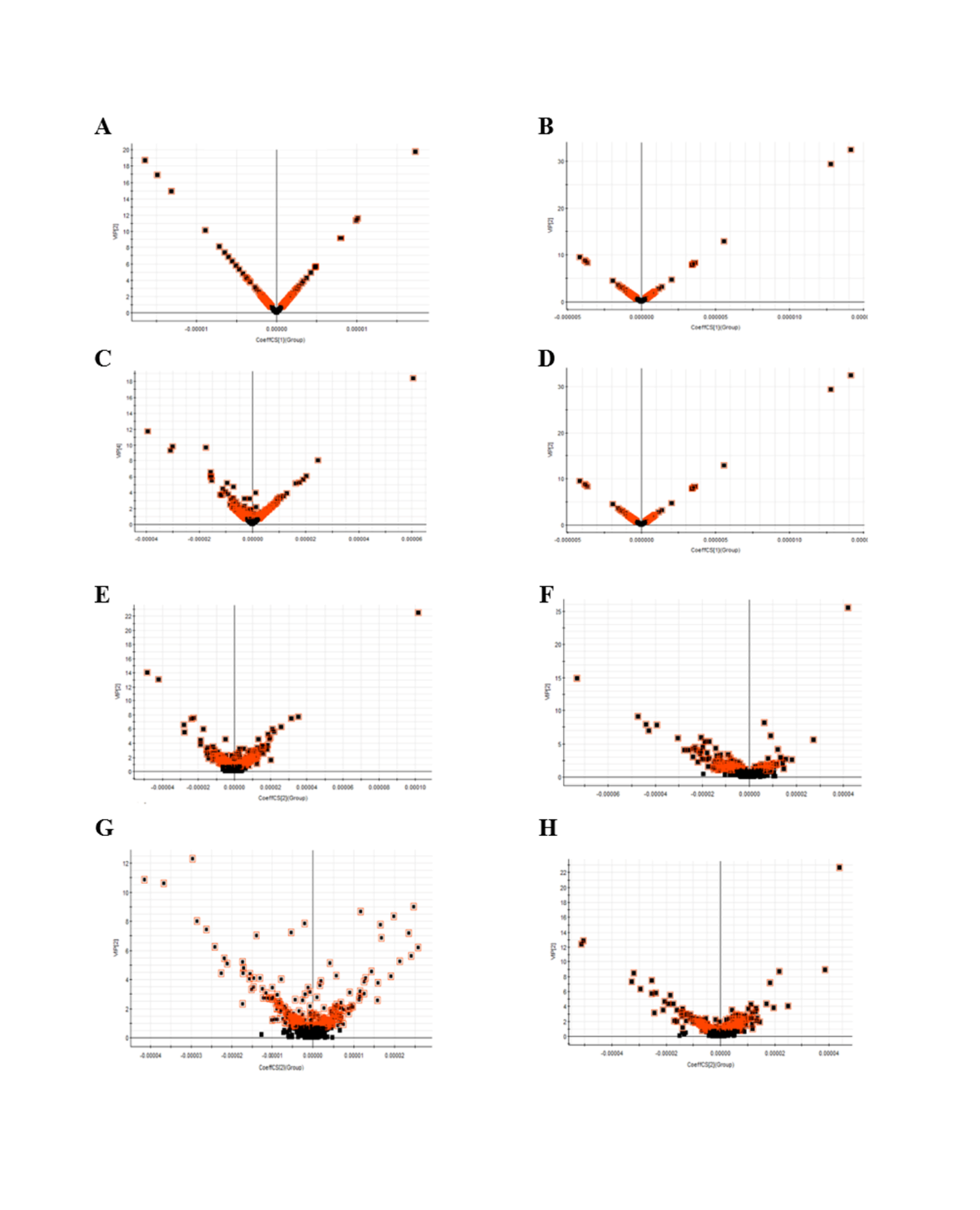
**

**Figure S6**


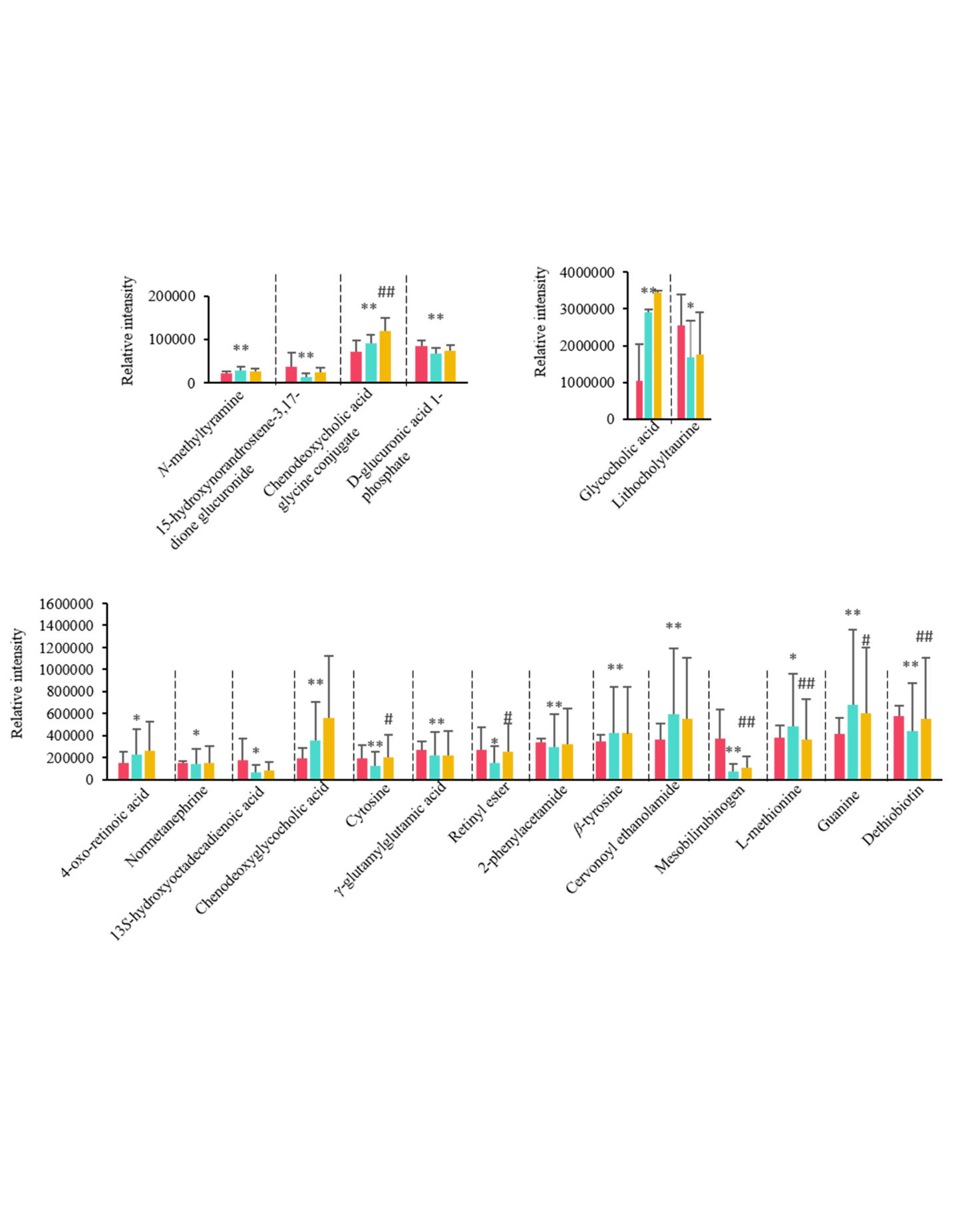


**Figure S7**


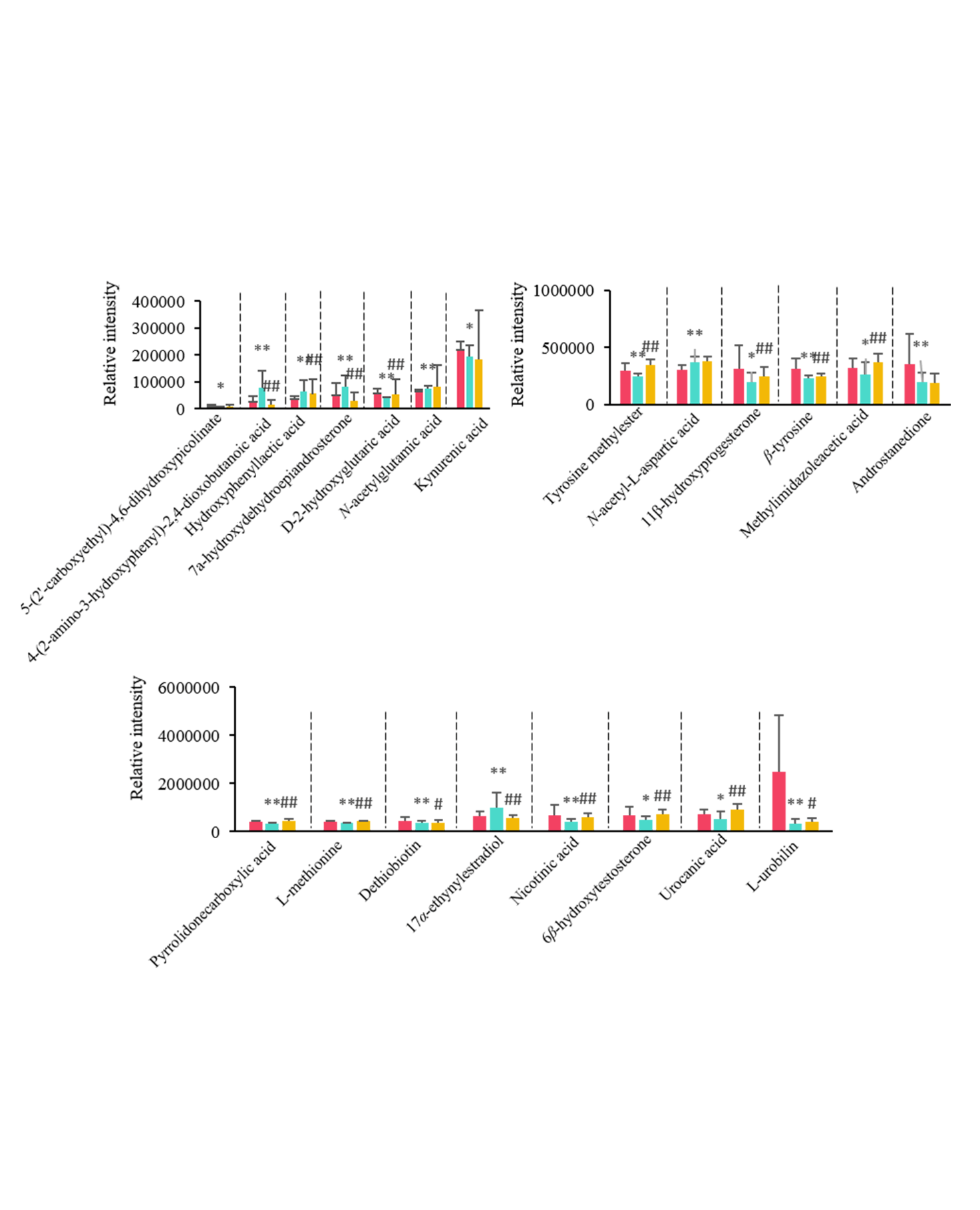


**Figure S8**


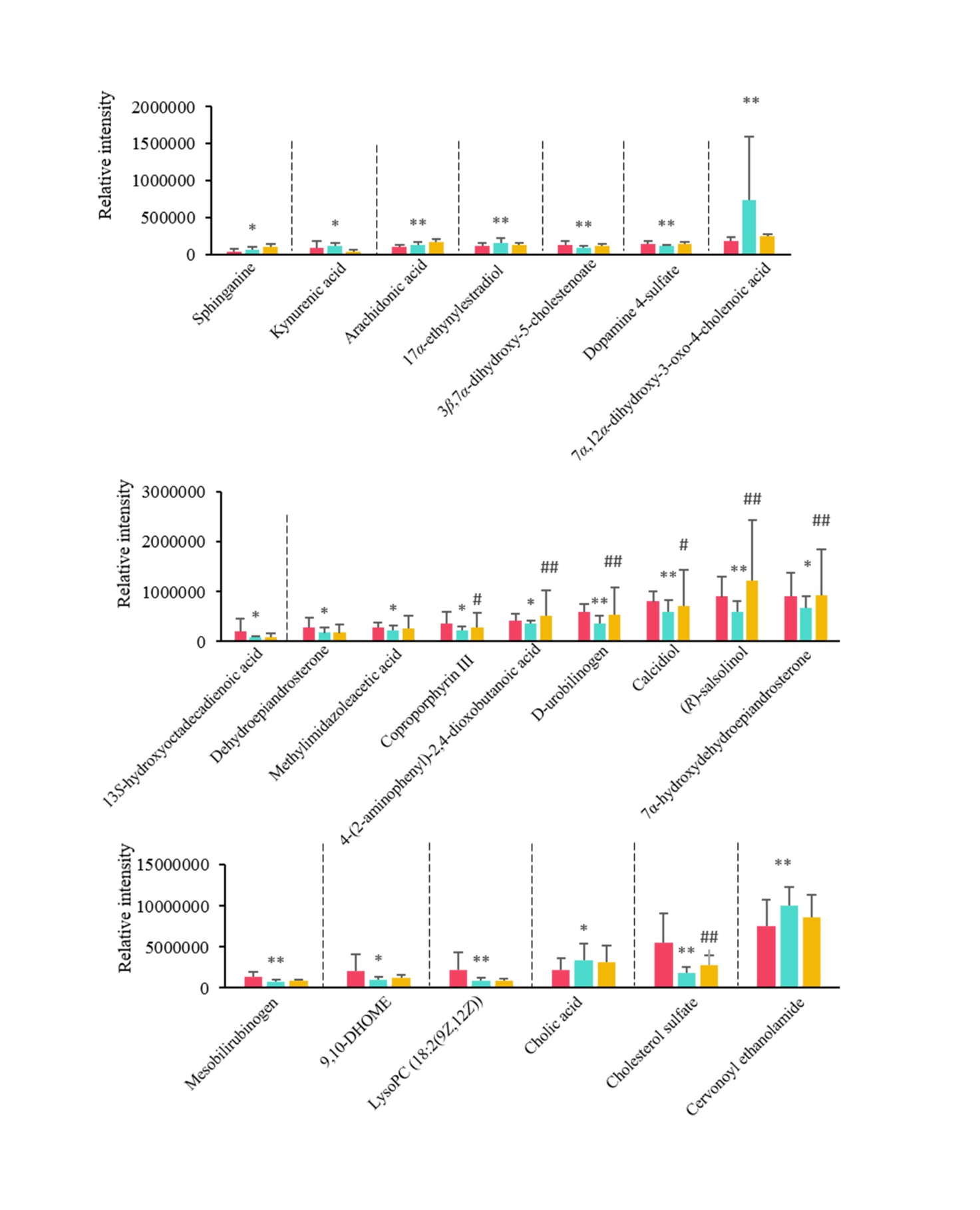


**Figure S9**

**
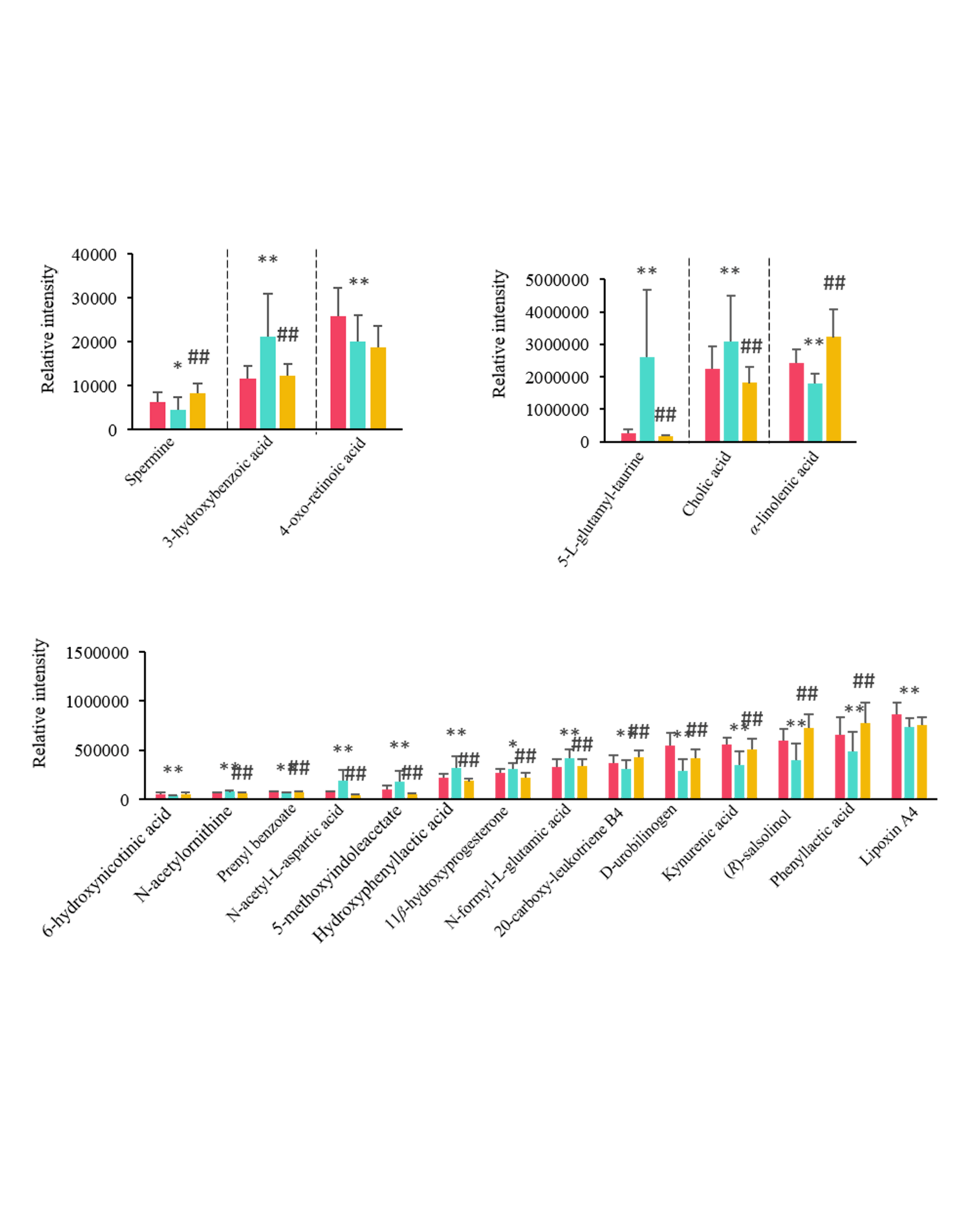
**

**Figure S10**


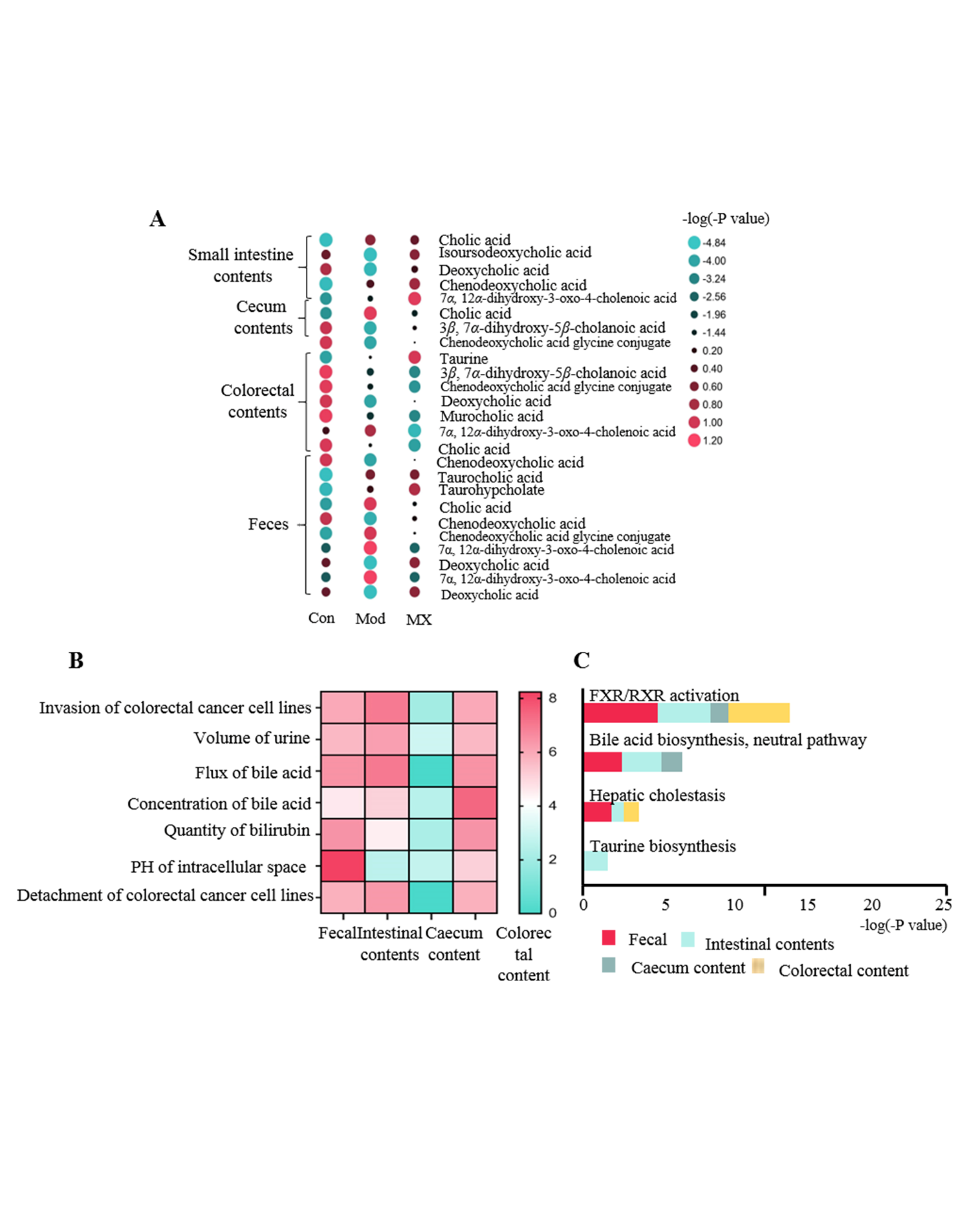


**Figure S11**


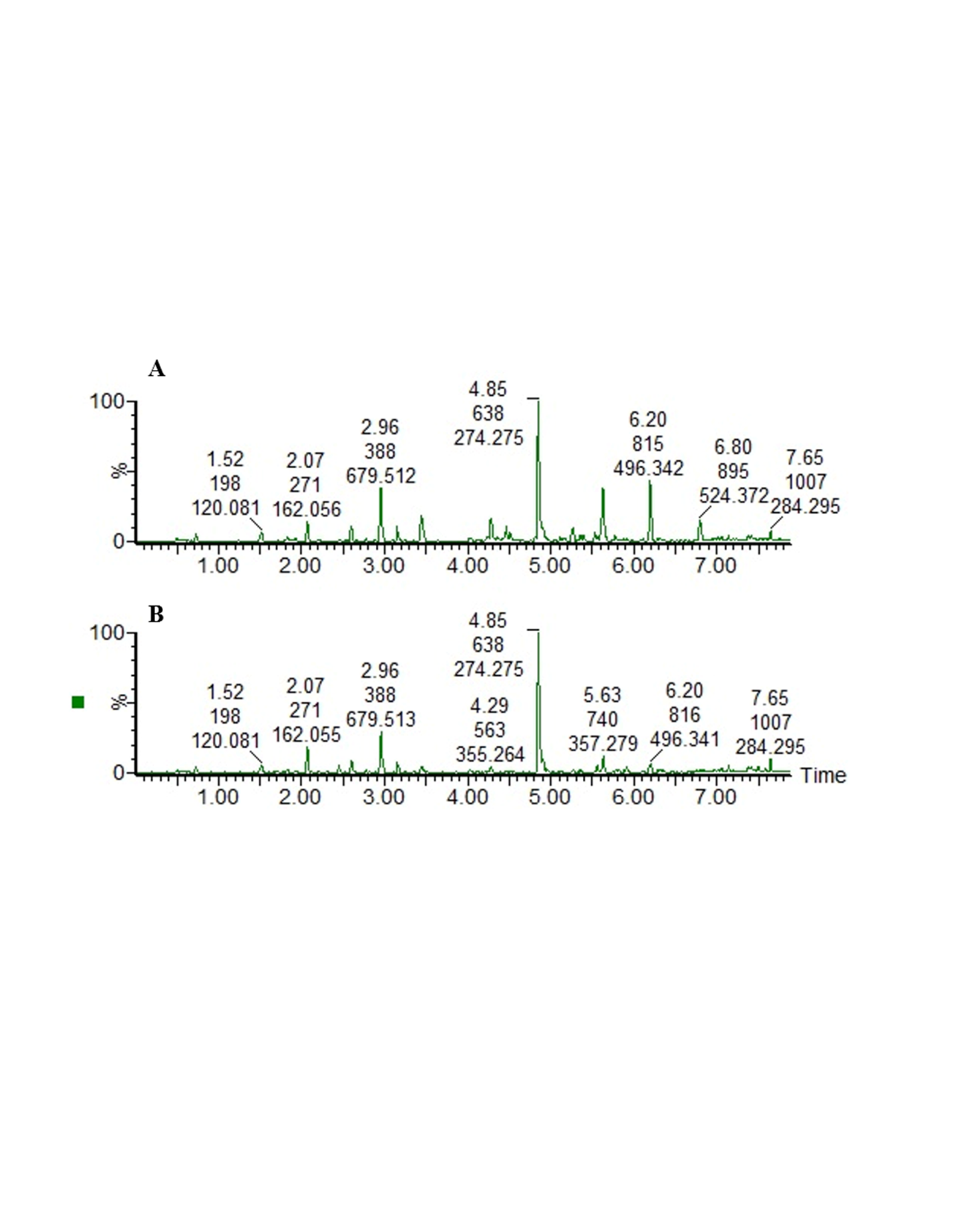


**Figure S12**


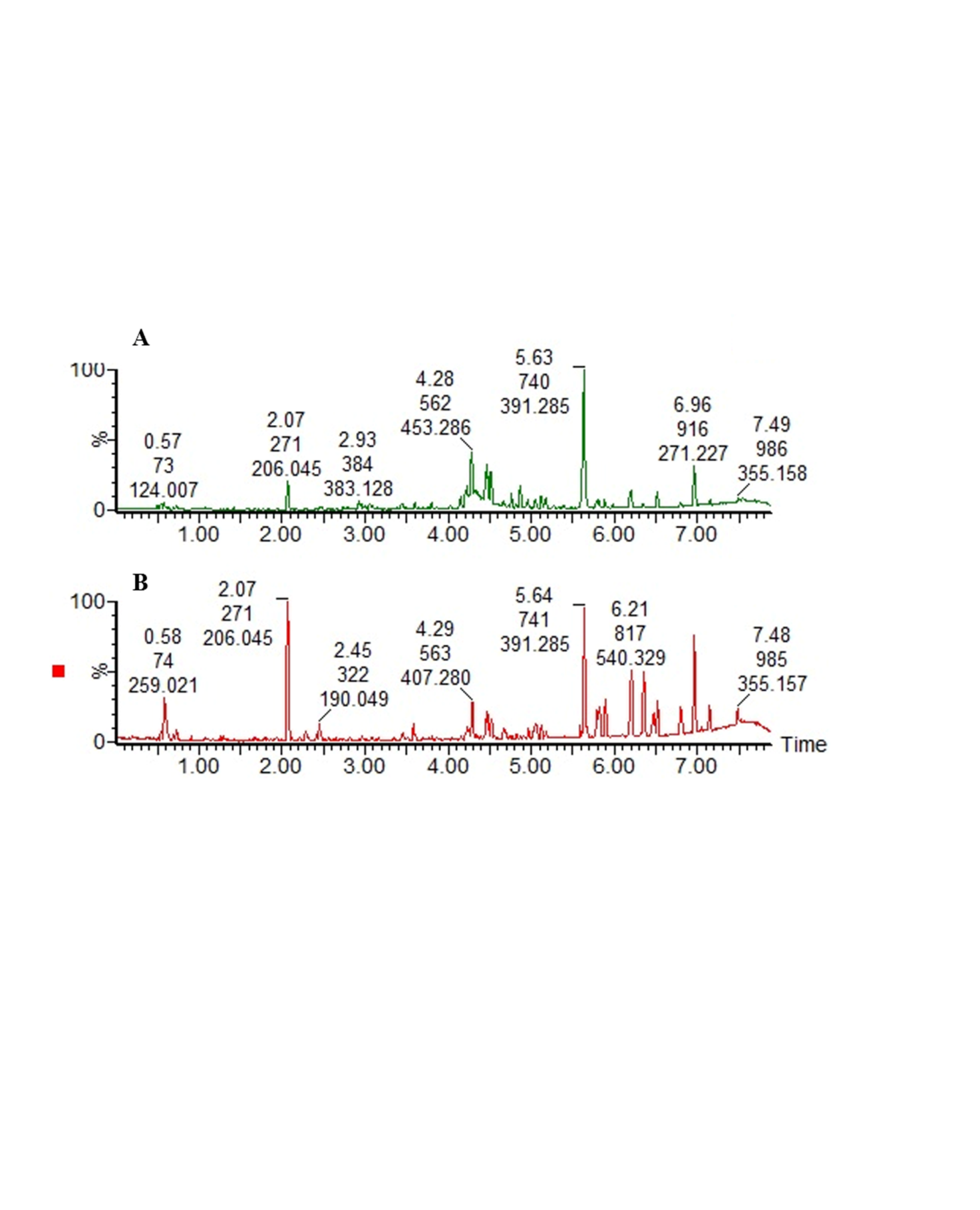


**Figure S13**


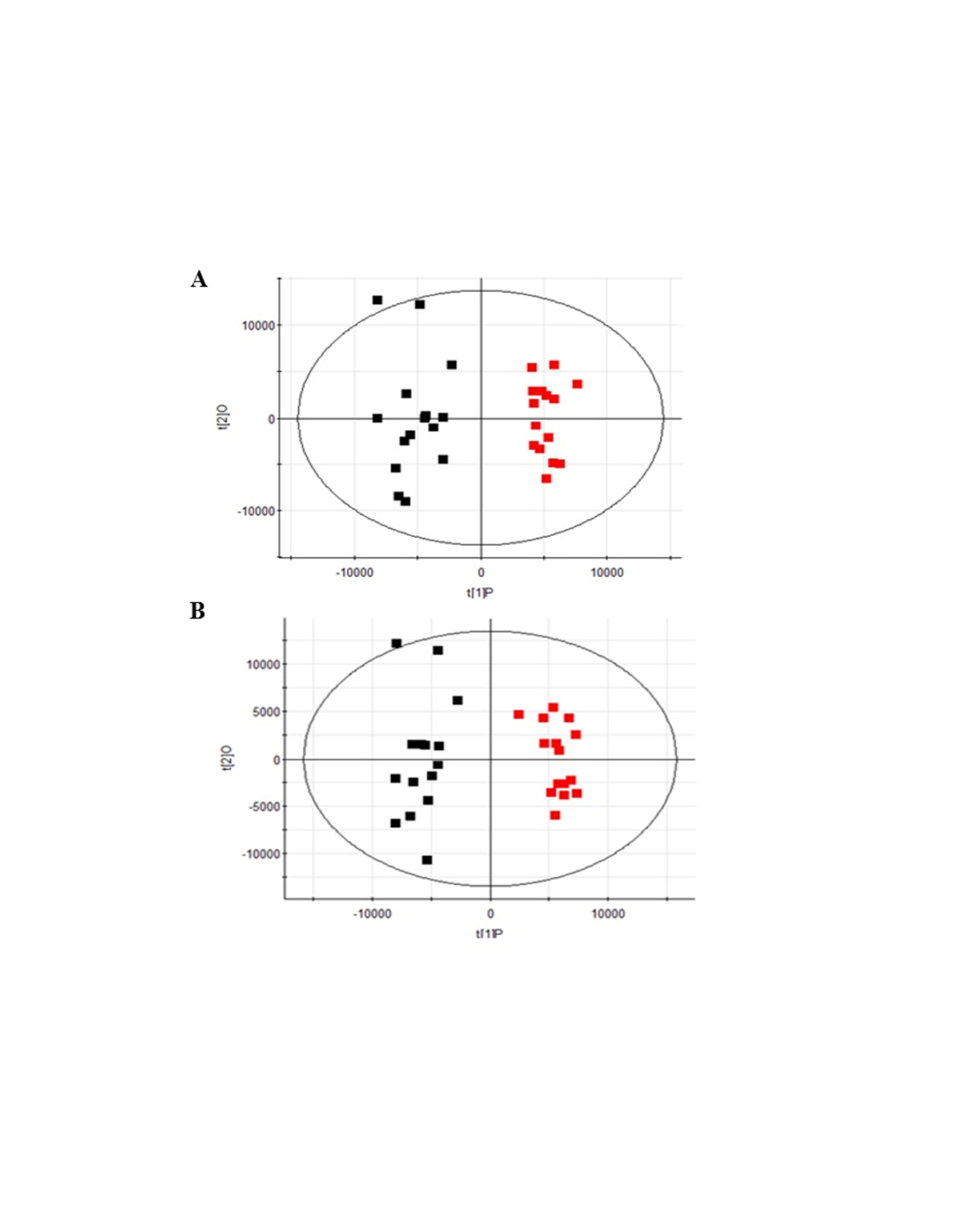


**Figure S14**


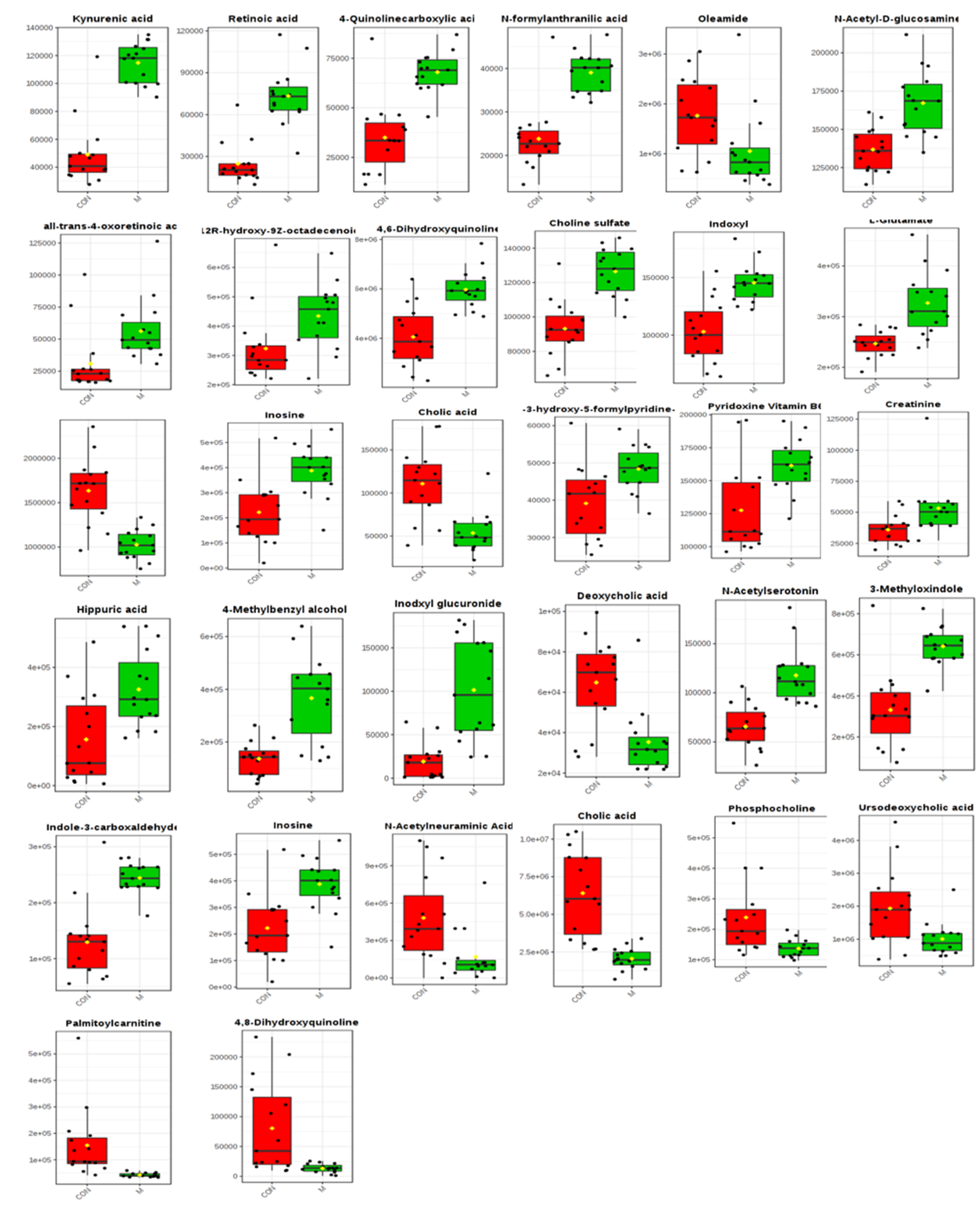


**Figure S15**


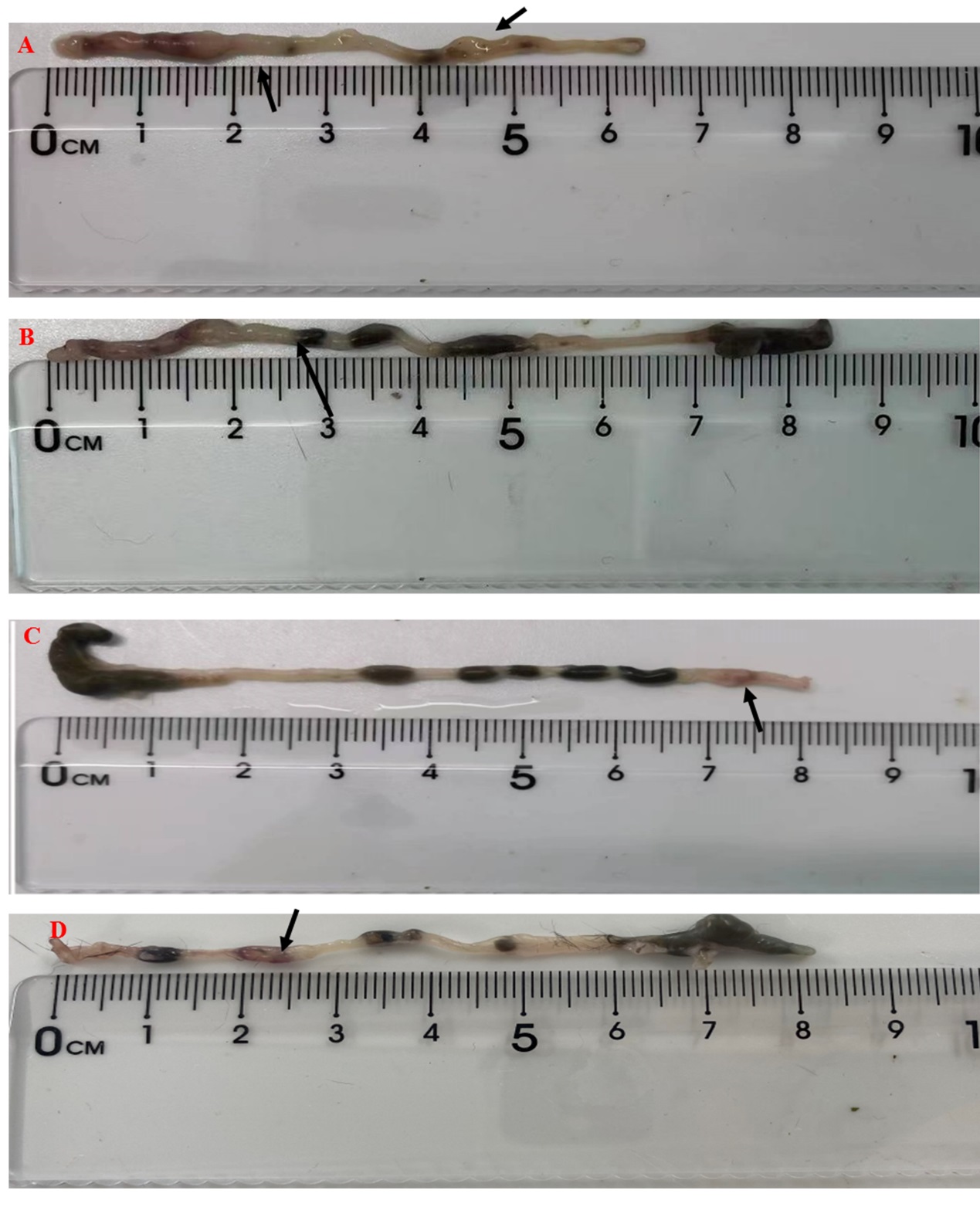


**Figure S16**

.


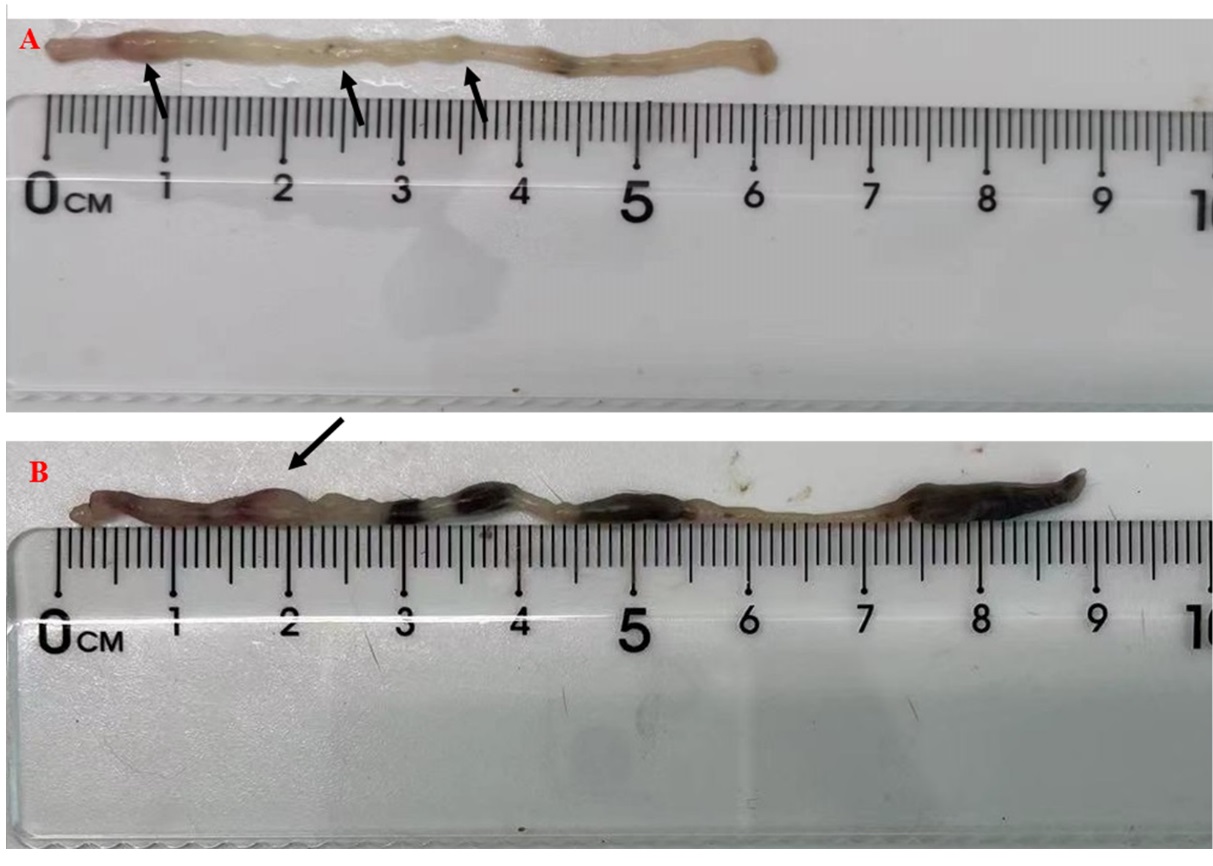


**Figure S17**


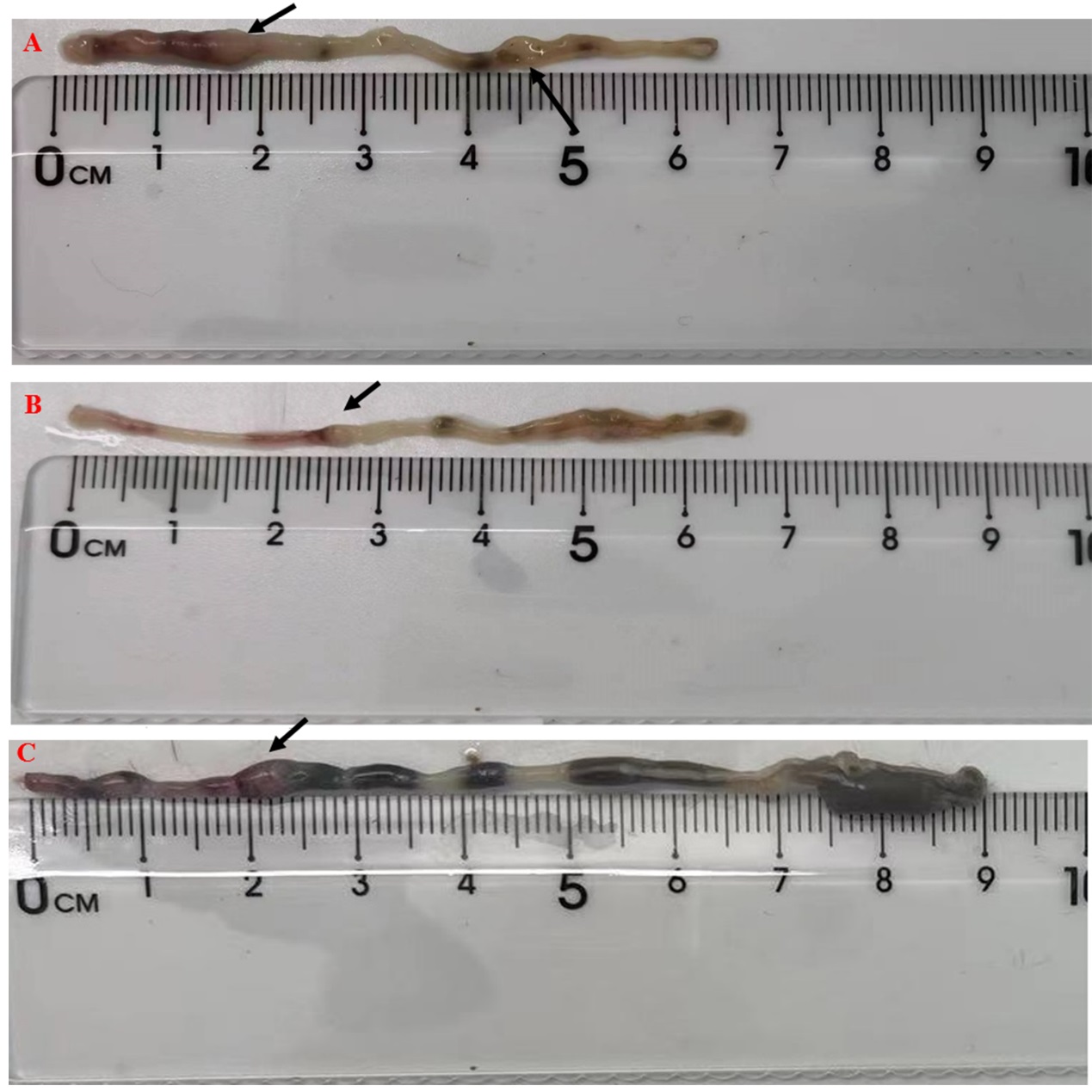


**Figure S18**

**Table S1** Biomarkers of serum in a model of intestinal tumors in positive mode

| No. | Retention time | *m/z* determined | Error (ppm) | Molecular formula | Ion form | Metabolite name | HMDB | KEGG | MX treatment |
| --- | --- | --- | --- | --- | --- | --- | --- | --- | --- |
| 1 | 0.75 | 132.0769 | 1.319227 | C_4_H_9_N_3_O_2_ | [M+H]^+^ | Beta-Guanidinopropionic acid | HMDB13222 | C03065 | ↑ |
| 2 | 1.12 | 153.0408 | 0.875783 | C_5_H_4_N_4_O_2_ | [M+H]^+^ | Xanthine | HMDB00292 | C00385 | - |
| 3 | 1.26 | 348.0705 | 0.310233 | C_10_H_14_N_5_O_7_P | [M+H]^+^ | Adenosine 2'-phosphate | HMDB11617 | C00946 | ↑ |
| 4 | 1.38 | 169.0358 | 0.821894 | C_5_H_4_N_4_O_3_ | [M+H]^+^ | Uric acid | HMDB0000289 | C00366 | ↓ |
| 5 | 1.52 | 132.1018 | -0.45464 | C_6_H_13_NO_2_ | [M+H]^+^ | L-Norleucine | HMDB01645 | C01933 | ↑ |
| 6 | 1.64 | 160.0759 | 1.087511 | C_10_H_9_NO | [M+H]^+^ | Indoleacetaldehyde | HMDB0001190 | C00637 | - |
| 7 | 2.27 | 118.0653 | 2.089989 | C_9_H_7_NO | [M+H]^+^ | Indole | HMDB00738 | C00463 | ↑ |
| 8 | 2.27 | 132.0811 | 2.098532 | C_9_H_9_N | [M+H]^+^ | 3-Methylindole | HMDB0000466 | C08313 | ↑ |
| 9 | 3.47 | 206.0814 | 0.934595 | C_11_H_11_NO_3_ | [M+H]^+^ | 5-Methoxyindoleacetate | HMDB0004096 | C05660 | ↑ |
| 10 | 4.12 | 333.2047 | -4.01002 | C_20_H_28_O_4_ | [M+H]^+^ | 11b-Hydroxyprogesterone | HMDB0004031 | C05498 | ↑ |
| 11 | 4.36 | 347.2211 | -1.63439 | C_21_H_30_O_4_ | [M+H]^+^ | Corticosterone | HMDB0001547 | C02140 | ↑ |
| 12 | 4.62 | 351.2174 | 2.347371 | C_20_H_30_O_5_ | [M+H]^+^ | Prostaglandin E3 | HMDB02664 | C06439 | ↑ |
| 13 | 6.04 | 544.3406 | 1.462988 | C_28_H_50_NO_7_P | [M+H]^+^ | LysoPC(20:4(5*Z*,8*Z*,11*Z*,14*Z*)) | HMDB0010395 | C04230 | ↓ |
| 14 | 9.89 | 329.2473 | -0.47749 | C_22_H_32_O_2_ | [M+H]^+^ | Docosahexaenoic acid | HMDB0002183 | C06429 | ↓ |

**Table S2** Biomarkers of serum in a model of intestinal tumors in negative mode

| No. | Retention time | *m/z* determined | Error (ppm) | Molecular formula | Ion form | Metabolite name | HMDB | KEGG | MX treatment |
| --- | --- | --- | --- | --- | --- | --- | --- | --- | --- |
| 1 | 1.01 | 346.0549 | -2.55268 | C_10_H_14_N_5_O_7_P | [M–H]^-^ | Adenosine monophosphate | HMDB0000045 | C00020 | ↑ |
| 2 | 1.8 | 180.0665 | -0.50175 | C_9_H_11_NO_3_ | [M–H]^-^ | Beta-Tyrosine | HMDB0003831 | C04368 | ↑ |
| 3 | 13.42 | 259.0214 | -3.84184 | C_6_H_13_O_9_P | [M–H]^-^ | Myo-inositol 1-phosphate | HMDB0000213 | C04006 | ↓ |
| 4 | 2.27 | 159.0927 | -0.56604 | C_10_H_12_N_2_ | [M–H]^-^ | Tryptamine | HMDB0000303) | C00398 | ↑ |
| 5 | 2.27 | 181.0508 | 0.719047 | C_9_H_10_O_4_ | [M–H]^-^ | Hydroxyphenyllactic acid | HMDB0000755 | C03672 | ↑ |
| 6 | 2.4 | 156.0664 | -1.20373 | C_7_H_11_NO_3_ | [M–H]^-^ | Tiglylglycine | HMDB0000959 | | ↓ |
| 7 | 2.54 | 128.0352 | -0.95176 | C_5_H_7_NO_3_ | [M–H]^-^ | Pyroglutamic acid | HMDB0000267 | C01879 | ↑ |
| 8 | 2.54 | 293.1148 | 1.622216 | C_14_H_18_N_2_O_5_ | [M–H]^-^ | Tyrosyl-Hydroxyproline | HMDB0029106 | | ↑ |
| 9 | 3.11 | 201.1134 | 0.841887 | C_10_H_18_O_4_ | [M–H]^-^ | Sebacic acid | HMDB0000792 | C08277 | ↑ |
| 10 | 3.4 | 107.0502 | -0.73436 | C_7_H_8_O | [M–H]^-^ | p-Cresol | HMDB0001858 | C01468 | ↓ |
| 11 | 3.47 | 204.0666 | 0.080019 | C_11_H_11_NO_3_ | [M–H]^-^ | Indolelactic acid | HMDB0000671 | C02043 | ↑ |
| 12 | 4.45 | 229.1443 | -1.06247 | C_12_H_22_O_4_ | [M–H]^-^ | Dodecanedioic acid | HMDB0000623 | C02678 | ↑ |
| 13 | 4.68 | 353.2317 | -4.76334 | C_20_H_34_O_5_ | [M–H]^-^ | Prostaglandin F2b | HMDB0001483 | C02314 | ↓ |
| 14 | 4.71 | 345.1707 | -0.04658 | C_20_H_26_O_5_ | [M–H]^-^ | 19-Noraldosterone | HMDB0041795 | | ↑ |
| 15 | 6.7 | 301.2174 | 0.267147 | C_20_H_30_O_2_ | [M–H]^-^ | Eicosapentaenoic acid | HMDB0001999 | C06428 | - |
| 16 | 9.25 | 301.2172 | -0.20568 | C_20_H_30_O_2_ | [M–H]^-^ | Retinyl ester | HMDB0003598 | C02075 | ↓ |

**Table S3** Biomarkers of small intestine contents in a model of intestinal tumors in positive mode

| No. | Retention  time | *m/z* determined | *m/z* calculated | Error (ppm) | Molecular formula | Ion form | Metabolite name | HMDB | KEGG |
| --- | --- | --- | --- | --- | --- | --- | --- | --- | --- |
| 1 | 0.67 | 112.0505 | 112.0511 | -0.6 | C_4_H_5_N_3_O | [M+H]^+^ | Cytosine | HMDB0000630 | C00380 |
| 2 | 0.69 | 152.057 | 152.0572 | 2.2 | C_5_H_5_N_5_O | [M+H]^+^ | Guanine | HMDB0000132 | C00242 |
| 3 | 1.44 | 150.0584 | 150.0589 | 0.4 | C_5_H_11_NO_2_S | [M+H]^+^ | L-Methionine | HMDB0000696 | C00073 |
| 4 | 1.47 | 182.081 | 182.0817 | 0.1 | C_9_H_11_NO_3_ | [M+H]^+^ | β-tyrosine | HMDB0003831 | C04368 |
| 5 | 1.55 | 184.0966 | 184.0974 | -0.9 | C_9_H_13_NO_3_ | [M+H]^+^ | Normetanephrine | HMDB0000819 | C05589 |
| 6 | 1.61 | 215.1387 | 215.1396 | -1.6 | C_10_H_18_N_2_O_3_ | [M+H]^+^ | Dethiobiotin | HMDB0003581 | C01909 |
| 7 | 1.66 | 136.0753 | 136.0762 | -3 | C_8_H_9_NO | [M+H]^+^ | 2-Phenylacetamide | HMDB0010715 | C02505 |
| 8 | 1.87 | 152.107 | 152.1075 | 0.06 | C_9_H_13_NO | [M+H]^+^ | *N*-methyltyramine | HMDB0003633 | C02442 |
| 9 | 4.65 | 315.1958 | 315.196 | -1.4 | C_20_H_26_O_3_ | [M+H]^+^ | 4-oxo-retinoic acid | HMDB0006285 | C16678 |
| 11 | 4.75 | 373.2746 | 373.2743 | 2.4 | C_24_H_36_O_3_ | [M+H]^+^ | Cervonoylethanolamide | HMDB0013627 |  |
| 10 | 4.8 | 593.3349 | 593.3339 | 3.8 | C_33_H_44_N_4_O_6_ | [M+H]^+^ | Mesobilirubinogen | HMDB0001898 | C05790 |

**Table S4** Biomarkers of small intestine contents in a model of intestinal tumors in negative mode

| No. | Retention time | *m/z* determined | *m/z* calculated | Error (ppm) | Molecular formula | Ion form | Metabolite name | HMDB | KEGG |
| --- | --- | --- | --- | --- | --- | --- | --- | --- | --- |
| 1 | 0.68 | 275.0881 | 275.0879 | -1.3 | C_10_H_16_N_2_O_7_ | [M–H]^-^ | γ-Glutamylglutamic acid | HMDB0011737 | C05282 |
| 2 | 1.8 | 312.1596 | 273.0012 | -2.1 | C_6_H_11_O_10_P | [M–H]^-^ | D-glucuronic acid 1-phosphate | HMDB0003976 | C05385 |
| 3 | 4.2 | 464.3017 | 464.3012 | 0 | C_26_H_43_NO_6_ | [M–H]^-^ | Glycocholic acid | HMDB0000138 | C01921 |
| 4 | 4.26 | 463.1986 | 463.1968 | 2.7 | C_24_H_32_O_9_ | [M–H]^-^ | 15-hydroxynorandrostene-3,17-dione glucuronide | HMDB0010353 | C03033 |
| 5 | 4.82 | 448.3058 | 448.3063 | -2.2 | C_26_H_43_NO_5_ | [M–H]^-^ | Chenodeoxycholic acid glycine conjugate | HMDB0000637 | C05466 |
| 6 | 4.94 | 448.3061 | 448.3063 | -1.5 | C_26_H_43_NO_5_ | [M–H]^-^ | Chenodeoxyglycocholic acid | HMDB0006898 | C05462 |
| 7 | 5.23 | 482.293 | 482.294 | -3.3 | C_26_H_45_NO_5_S | [M–H]^-^ | Lithocholyltaurine | HMDB0000722 | C02592 |
| 8 | 5.89 | 295.2267 | 295.2273 | -3.8 | C_18_H_32_O_3_ | [M–H]^-^ | 13*S*-hydroxyoctadecadienoic acid | HMDB0004667 | C14762 |
| 9 | 6.66 | 301.216 | 301.2168 | -4.2 | C_20_H_30_O_2_ | [M–H]^-^ | Retinyl ester | HMDB0003598 | C02075 |

**Table S5** Biomarkers ofcecal contents in a model of intestinal tumors in positive mode

| NO | RT | m/z determine | m/z calculated | Error  (ppm) | Molecular Formula | Ionform | Metabolite Name | HMDB | KEGG |
| --- | --- | --- | --- | --- | --- | --- | --- | --- | --- |
| 1 | 0.58 | 130.0498 | 130.0504 | -0.5 | C_5_H_7_NO_3_ | [M+H]+ | Pyrrolidonecarboxylic acid | HMDB0000805 | C02237 |
| 2 | 0.59 | 141.0658 | 141.0664 | -0.6 | C_6_H_8_N_2_O_2_ | [M+H]+ | Methylimidazoleacetic acid | HMDB0002820 | C05828 |
| 3 | 0.68 | 139.0504 | 139.0508 | 1.4 | C_6_H_6_N_2_O_2_ | [M+H]+ | Urocanic acid | HMDB0000301 | C00785 |
| 4 | 0.69 | 124.0396 | 124.0399 | 2.2 | C_6_H_5_NO_2_ | [M+H]+ | Nicotinic acid | HMDB0001488 | C00253 |
| 5 | 1.19 | 196.0977 | 196.0974 | 4.2 | C_10_H_13_NO_3_ | [M+H]+ | Tyrosine methylester | HMDB0029217 | C03404 |
| 6 | 1.34 | 182.0817 | 182.0817 | 2.7 | C_9_H_11_NO_3_ | [M+H]+ | Beta-Tyrosine | HMDB0003831 | C04368 |
| 7 | 1.5 | 228.0511 | 228.0508 | 3.5 | C_9_H_9_NO_6_ | [M+H]+ | 5-(2'-Carboxyethyl)-4,6-Dihydroxypicolinate | HMDB0006794 | C05655 |
| 8 | 2.14 | 150.0585 | 150.0589 | 1.2 | C_5_H_11_NO_2_S | [M+H]+ | L-Methionine | HMDB0000696 | C00073 |
| 9 | 2.44 | 215.1389 | 215.1396 | -0.6 | C_10_H_18_N_2_O_3_ | [M+H]+ | Dethiobiotin | HMDB0003581 | C01909 |
| 10 | 4.31 | 297.1855 | 297.1855 | -1.6 | C_20_H_24_O_2_ | [M+H]+ | 17a-Ethynylestradiol | HMDB0001926 | C07534 |
| 11 | 4.74 | 595.3484 | 595.3496 | -1 | C_33_H_46_N_4_O_6_ | [M+H]+ | L-Urobilin | HMDB0004159 | C05793 |
| 12 | 4.76 | 333.2067 | 333.2066 | 2 | C_20_H_28_O_4_ | [M+H]+ | 11b-Hydroxyprogesterone | HMDB0004031 | C05498 |

**Table S6** Biomarkers ofcecal contents in a model of intestinal tumors in negative model

| No. | Retention time | *m/z* determined | *m/z* calculated | Error  (ppm) | Molecular formula | Ion form | Metabolite name | HMDB | KEGG |
| --- | --- | --- | --- | --- | --- | --- | --- | --- | --- |
| 1 | 0.7 | 147.0293 | 147.0293 | -4.2 | C_5_H_8_O_5_ | [M–H]^-^ | D-2-hydroxyglutaric acid | HMDB0000606 | C01087 |
| 2 | 0.7 | 174.0401 | 174.0402 | -4.1 | C_6_H_9_NO_5_ | [M–H]^-^ | *N*-acetyl-L-aspartic acid | HMDB0000812 | C01042 |
| 3 | 0.71 | 188.0557 | 188.0559 | -3.8 | C_7_H_11_NO_5_ | [M–H]^-^ | *N*-acetylglutamic acid | HMDB0001138 | C00624 |
| 4 | 1.41 | 222.0398 | 222.0402 | -4.6 | C_10_H_9_NO_5_ | [M–H]^-^ | 4-(2-amino-3-hydroxyphenyl)-2,4-dioxobutanoic acid | HMDB04083 | C05645 |
| 5 | 1.78 | 181.0515 | 181.0501 | 4.8 | C_9_H_10_O_4_ | [M–H]^-^ | Hydroxyphenyllactic acid | HMDB00755 | C03672 |
| 6 | 2.01 | 188.0349 | 188.0348 | -2 | C_10_H_7_NO_3_ | [M–H]^-^ | Kynurenic acid | HMDB00715 | C01717 |
| 7 | 4.36 | 303.1956 | 303.196 | -3.2 | C_19_H_28_O_3_ | [M–H]^-^ | 6*β*-hydroxytestosterone | HMDB06259 | C14497 |
| 8 | 4.36 | 303.1956 | 303.196 | -3.2 | C_19_H_28_O_3_ | [M–H]^-^ | 7*α*-hydroxydehydroepiandrosterone | HMDB04611 | C18045 |
| 9 | 5.09 | 287.2009 | 287.2011 | -2.7 | C_19_H_28_O_2_ | [M–H]^-^ | Androstanedione | HMDB0000899 | C00674 |

**Table S7** Biomarkers of colorectal contents in a model of intestinal tumors in positive mode

| No. | Retention time | *m/z* determined | *m/z* calculated | Error (ppm) | Molecular formula | Ion form | Metabolite name | HMDB | KEGG |
| --- | --- | --- | --- | --- | --- | --- | --- | --- | --- |
| 1 | 0.58 | 141.0659 | 141.0664 | 0.6 | C_6_H_8_N_2_O_2_ | [M+H]^+^ | Methylimidazoleacetic acid | HMDB0002820 | C05828 |
| 2 | 2.95 | 180.1018 | 180.1025 | -0.3 | C_10_H_13_NO_2_ | [M+H]^+^ | (*R*)-Salsolinol | HMDB0005199 | C09642 |
| 3 | 3.5 | 297.1847 | 297.1855 | 2.4 | C_20_H_24_O_2_ | [M+H]^+^ | 17*α*-ethynylestradiol | HMDB0001926 | C07534 |
| 4 | 4.08 | 405.2646 | 405.2641 | 2.8 | C_24_H_36_O_5_ | [M+H]^+^ | 7a,12a-dihydroxy-3-oxo-4-cholenoic acid | HMDB0000447 | C15568 |
| 5 | 4.12 | 373.2749 | 373.2743 | 3 | C_24_H_36_O_3_ | [M+H]^+^ | Cervonoylethanolamide | HMDB0013627 | C13828 |
| 6 | 4.26 | 655.2788 | 655.2768 | 3.9 | C_36_H_38_N_4_O_8_ | [M+H]^+^ | Coproporphyrin III | HMDB0000570 | C05770 |
| 7 | 5.51 | 302.3039 | 301.2168 | 0.4 | C_18_H_39_NO_2_ | [M+H]^+^ | Sphinganine | HMDB0000269 | C00836 |
| 8 | 5.78 | 520.3405 | 520.3403 | 1.3 | C_26_H_50_NO_7_P | [M+H]^+^ | LysoPC(18:2(9*Z*,12*Z*)) | HMDB0010386 |  |
| 9 | 6.89 | 305.2477 | 305.2481 | 0.6 | C_20_H_32_O_2_ | [M+H]^+^ | Arachidonic acid | HMDB0001043 | C00219 |
| 10 | 7.02 | 401.3404 | 401.342 | -2.5 | C_27_H_44_O_2_ | [M+H]^+^ | Calcidiol | HMDB0003550 | C01561 |

**Table S8** Biomarkers of colorectal contents in a model of intestinal tumors in negative mode

| No. | Retention time | *m/z* determined | *m/z* calculated | Error (ppm) | Molecular formula | Ion form | Metabolite name | HMDB | KEGG |
| --- | --- | --- | --- | --- | --- | --- | --- | --- | --- |
| 1 | 1.48 | 232.029 | 232.028 | 2.1 | C_8_H_11_NO_5_S | [M­–H]^-^ | Dopamine 4-sulfate | HMDB0004148 | C13691 |
| 2 | 1.98 | 206.0449 | 206.0453 | -4.7 | C_10_H_9_NO_4_ | [M–H]^-^ | 4-(2-aminophenyl)-2,4-dioxobutanoic acid | HMDB0000978 | C01252 |
| 3 | 1.98 | 188.0349 | 188.0348 | -2 | C_10_H_7_NO_3_ | [M–H]^-^ | Kynurenic acid | HMDB0000715 | C01717 |
| 4 | 3.33 | 589.3007 | 591.3183 | -4.2 | C_33_H_42_N_4_O_6_ | [M–H]^-^ | D-Urobilinogen | HMDB04158 | C05791 |
| 5 | 3.98 | 407.2801 | 407.2797 | 0.9 | C_24_H_40_O_5_ | [M–H]^-^ | Cholic acid | HMDB0000619 | C00695 |
| 6 | 4.34 | 303.1955 | 303.196 | -3.5 | C_19_H_28_O_3_ | [M–H]^-^ | 7*α*-hydroxydehydroepiandrosterone | HMDB04611 | C18045 |
|  | 4.76 | 591.3213 | 591.3183 | 4.2 | C_33_H_44_N_4_O_6_ | [M–H]^-^ | Mesobilirubinogen | HMDB01898 | C05790 |
| 8 | 4.98 | 313.2375 | 313.2379 | -3 | C_18_H_34_O_4_ | [M–H]^-^ | 9,10-DHOME | HMDB0004704 | C14828 |
| 9 | 5.06 | 287.2006 | 287.2011 | -3.1 | C_19_H_28_O_2_ | [M–H]^-^ | Dehydroepiandrosterone | HMDB0000077 | C01227 |
| 10 | 5.83 | 295.2268 | 295.2273 | -3.5 | C_18_H_32_O_3_ | [M–H]^-^ | 13*S*-hydroxyoctadecadienoic acid | HMDB0004667 | C14762 |
| 11 | 6.95 | 431.3161 | 431.3161 | -1.3 | C_27_H_44_O_4_ | [M–H]^-^ | 3*β*,7*α*-dihydroxy-5-cholestenoate | HMDB0012454 | C17335 |
| 12 | 9.07 | 465.3036 | 465.3039 | -1.7 | C_27_H_46_O_4_S | [M–H]^-^ | Cholesterol sulfate | HMDB0000653 | C18043 |

**Table S9** Biomarkers of feces in a model of intestinal tumors in positive mode

| No. | Retention time | *m/z* determined | *m/z* calculated | Error (ppm) | Molecular formula | Ion form | Metabolite name | HMDB | KEGG |
| --- | --- | --- | --- | --- | --- | --- | --- | --- | --- |
| 1 | 1.2 | 140.0344 | 140.0348 | 1 | C_6_H_5_NO_3_ | [M+H]^+^ | 6-Hydroxynicotinic acid | HMDB0002658 | C01020 |
| 2 | 1.84 | 203.2228 | 203.2236 | -1.2 | C_10_H_26_N_4_ | [M+H]^+^ | Spermine | HMDB0001256 | C00750 |
| 3 | 1.87 | 190.0502 | 190.0504 | 1.8 | C_10_H_7_NO_3_ | [M+H]^+^ | Kynurenic acid | HMDB0000715 | C01717 |
| 4 | 2.4 | 191.1065 | 191.1072 | -0.6 | C_12_H_14_O_2_ | [M+H]^+^ | Prenyl benzoate | HMDB0032488 | C03885 |
| 5 | 2.92 | 180.1014 | 180.1025 | -2.5 | C_10_H_13_NO_2_ | [M+H]^+^ | (*R*)-salsolinol | HMDB0005199 | C09642 |
| 6 | 3.36 | 591.3185 | 591.3183 | 1.2 | C_33_H_42_N_4_O_6_ | [M+H]^+^ | D-urobilinogen | HMDB0004158 | C05791 |
| 7 | 5.03 | 279.2318 | 279.2324 | -0.2 | C_18_H_30_O_2_ | [M+H]^+^ | *α*-linolenic acid | HMDB0001388 | C06427 |

**Table S10** Biomarkers of feces in a model of intestinal tumors model in negative mode

| No. | Retention time | *m/z* determined | *m/z* calculated | Error (ppm) | Molecular formula | Ion form | Metabolite name | HMDB | KEGG |
| --- | --- | --- | --- | --- | --- | --- | --- | --- | --- |
| 1 | 0.6 | 174.04 | 174.0402 | -4.7 | C_6_H_9_NO_5_ | [M–H]^-^ | *N*-Acetyl-L-aspartic acid | HMDB0000812 | C01042 |
| 2 | 0.63 | 173.0925 | 174.1004 | -4 | C_7_H_14_N_2_O_3_ | [M–H]^-^ | *N*-Acetylornithine | HMDB0003357 | C00437 |
| 3 | 0.7 | 174.04 | 174.0402 | -4.6 | C_6_H_9_NO_5_ | [M–H]^-^ | *N*-Formyl-L-glutamic acid | HMDB0003470 | C01045 |
| 4 | 1.79 | 181.0513 | 181.0501 | 3.5 | C_9_H_10_O_4_ | [M–H]^-^ | Hydroxyphenyllactic acid | HMDB00755 | C03672 |
| 5 | 1.98 | 137.0242 | 137.0239 | -1.4 | C_7_H_6_O_3_ | [M–H]^-^ | 3-Hydroxybenzoic acid | HMDB0002466 | C00587 |
| 6 | 2.65 | 165.0551 | 165.0552 | -3.5 | C_9_H_10_O_3_ | [M–H]^-^ | Phenyllactic acid | HMDB00779 | C01479 |
| 7 | 2.72 | 365.1957 | 365.1964 | -3.3 | C_20_H_30_O_6_ | [M–H]^-^ | 20-Carboxy-leukotriene B4 | HMDB0006059 | C05950 |
| 8 | 2.77 | 204.0656 | 204.0661 | -4.8 | C_11_H_11_NO_3_ | [M–H]^-^ | 5-Methoxyindoleacetate | HMDB0004096 | C05660 |
| 9 | 3.25 | 253.0504 | 253.0494 | 1.6 | C_7_H_14_N_2_O_6_S | [M–H]^-^ | 5-L-Glutamyl-taurine | HMDB04195 | C05844 |
| 10 | 3.5 | 351.2164 | 351.2171 | -3.6 | C_20_H_32_O_5_ | [M–H]^-^ | Lipoxin A4 | HMDB04385 | C06314 |
| 11 | 3.65 | 331.1899 | 331.1909 | -4.8 | C_20_H_28_O_4_ | [M–H]^-^ | 11b-Hydroxyprogesterone | HMDB0004031 | C05498 |
| 12 | 4.04 | 407.2796 | 407.2797 | -1.7 | C_24_H_40_O_5_ | [M-H]- | Cholic acid | HMDB00619 | C00695 |
| 13 | 5.55 | 313.1811 | 313.1804 | 0.5 | C_20_H_26_O_3_ | [M-H]- | 4-oxo-Retinoic acid | HMDB0006285 | C16678 |

**Table S11** MX influencing metabolite biomarkers of intestine contents in the intestinal tumor model

|  | MX treatment |
| --- | --- |
| γ-gGlutamylglutamic acid | - |
| D-glucuronic acid 1-phosphate | √ |
| Glycocholic acid | √ |
| 15-hydroxynorandrostene-3,17-dione glucuronide | √ |
| Chenodeoxycholic acid glycine conjugate | √ |
| Chenodeoxyglycocholic acid | √ |
| Lithocholyltaurine | - |
| 13*S*-hydroxyoctadecadienoic acid | √ |
| Retinyl ester | √ |
| Cytosine | - |
| Guanine | √ |
| L-Methionine | √ |
| β-Tyrosine | √ |
| Normetanephrine | √ |
| Dethiobiotin | √ |
| 2-phenylacetamide | - |
| *N*-methyltyramine | √ |
| Cervonoylethanolamide | - |
| 4-oxo-retinoic acid | √ |
| Mesobilirubinogen | √ |
|  | 15 |

**Table S12** MX influencing metabolite biomarkers of cecal contents

|  | MX treatment |
| --- | --- |
| D-2-hydroxyglutaric acid | √ |
| *N*-acetyl-L-aspartic acid | - |
| *N*-acetylglutamic acid | - |
| D-2-Hydroxyglutaric acid | √ |
| 4-(2-amino-3-hydroxyphenyl)-2,4-dioxobutanoic acid | √ |
| Hydroxyphenyllactic acid | √ |
| Kynurenic acid | √ |
| 7α-hydroxydehydroepiandrosterone | √ |
| 6*β*-Hydroxytestosterone | √ |
| Pyrrolidonecarboxylic acid | √ |
| Methylimidazoleacetic acid | √ |
| Urocanic acid | √ |
| Nicotinic acid | √ |
| Tyrosine methylester | √ |
| *β*-tyrosine | √ |
| 5-(2'-carboxyethyl)-4,6-dihydroxypicolinate | √ |
| L-methionine | √ |
| Dethiobiotin | √ |
| 17α-Ethynylestradiol | √ |
| L-urobilin | √ |
| 11*β*-Hydroxyprogesterone | √ |
|  | 19 |

**Table S13** Potential differentiating metabolites identified in MX-treated normal mouse in positive mode

| **No.** | **Retention time** | ***m/z* determined** | ***m/z* calculated** | **Error (ppm)** | **Molecular formula** | **Ion form** | **Actual_molecular weight** | **Metabolite name** | **KEGG** | **HMDB** |
| --- | --- | --- | --- | --- | --- | --- | --- | --- | --- | --- |
| 1 | 0.59 | 148.0613 | 148.0610 | 2.0 | C_5_H_9_NO_4_ | [M+H]^+^ | 147.0532 | L-glutamate | C00025 | HMDB00148 |
| 2 | 0.6 | 118.0863 | 118.0873 | 4.2 | C_5_H_11_NO_2_ | [M+H]^+^ | 117.0790 | L-valine | C00183 | HMDB00883 |
| 3 | 0.62 | 222.0981 | 222.0978 | 1.4 | C_8_H_15_NO_6_ | [M+H]^+^ | 221.0899 | *N*-acetyl-D-glucosamine | C00140 | HMDB00215 |
| 4 | 0.64 | 182.0449 | 182.0453 | -2.2 | C_8_H_7_NO_4_ | [M+H]^+^ | 181.0375 | 2-methyl-3-hydroxy-5-formylpyridine-4-carboxylate | C06050 | HMDB06954 |
| 5 | 0.65 | 114.0663 | 114.0667 | -3.5 | C_4_H_7_N_3_O | [M+H]^+^ | 113.0589 | Creatinine | C00791 | HMDB00562 |
| 6 | 0.7 | 184.0647 | 184.0644 | 1.6 | C_5_H_13_NO_4_S | [M+H]^+^ | 183.0565 | Choline sulfate | C00919 | - |
| 7 | 0.87 | 170.0815 | 170.0817 | -1.2 | C_8_H_11_NO_3_ | [M+H]^+^ | 169.0739 | Pyridoxine (vitamin B6) | C00314 | HMDB00239 |
| 8 | 1.29 | 166.0507 | 166.0504 | 1.8 | C_8_H_7_NO_3_ | [M+H]^+^ | 165.0426 | *N*-formylanthranilic acid | C05653 | HMDB04089 |
| 9 | 1.83 | 271.1666 | 271.1698 | -11.8 | C_18_H_22_O_2_ | [M+H]^+^ | 270.1620 | Estrone | C00468 | HMDB00145 |
| 10 | 2.07 | 162.0558 | 162.0555 | 1.9 | C_9_H_7_NO_2_ | [M+H]^+^ | 161.0477 | 4,6-dihydroxyquinoline | C05639 | HMDB04077 |
| 11 | 2.07 | 134.0603 | 134.0606 | -2.2 | C_8_H_7_NO | [M+H]^+^ | 133.0528 | Indoxyl | C05658 | HMDB04094 |
| 12 | 2.45 | 174.0556 | 174.0555 | 0.6 | C_10_H_7_NO_2_ | [M+H]^+^ | 173.0477 | 4-quinolinecarboxylic acid | C06414 | - |
| 13 | 3.15 | 190.0504 | 190.0504 | 0.0 | C_10_H_7_NO_3_ | [M+H]^+^ | 189.0426 | Kynurenic acid | C01717 | HMDB00715 |
| 14 | 4.28 | 409.2956 | 409.2954 | 0.5 | C_24_H_40_O_5_ | [M+H]^+^ | 408.2876 | Cholic acid | C00695 | HMDB00619 |
| 15 | 4.84 | 301.2172 | 301.2168 | 1.3 | C_20_H_28_O_2_ | [M+H]^+^ | 300.2089 | Retinoic acid | C00777 | HMDB12874 |
| 16 | 5.53 | 282.2798 | 282.2797 | 0.4 | C_18_H_35_NO | [M+H]^+^ | 281.2719 | Oleamide | C19670 | HMDB02117 |
| 17 | 5.66 | 393.3109 | 393.3109 | 0.0 | C_24_H_40_O_4_ | [M+H]^+^ | 392.2927 | Deoxycholic acid | C04483 | HMDB00626 |
| 18 | 5.85 | 315.1967 | 315.1960 | 2.2 | C_20_H_26_O_3_ | [M+H]^+^ | 314.1882 | all-trans-4-oxoretinoic acid | C16678 | HMDB06285 |
| 19 | 6.15 | 400.3432 | 400.3427 | 1.2 | C_23_H_45_NO_4_ | [M+H]^+^ | 399.3349 | Palmitoylcarnitine | C02990 | HMDB00222 |
| 20 | 6.2 | 184.0737 | 184.0739 | -1.1 | C_5_H_14_NO_4_P | [M+H]^+^ | 183.0660 | Phosphocholine | C00588 | HMDB01565 |

**Table S14** Potential differentiating metabolites identified in MX-treated normal mouse in negative mode

| **No.** | **Retention time** | ***m/z* determined** | ***m/z* calculated** | **Error (ppm)** | **Molecular Formula** | **Ion form** | **Actual molecular weight** | **Metabolite name** | **KEGG** | **HMDB** |
| --- | --- | --- | --- | --- | --- | --- | --- | --- | --- | --- |
| 1 | 0.62 | 160.0372 | 160.0399 | -16.9 | C_9_H_7_NO_2_ | [M­–H]- | 161.0477 | 4,8-dihydroxyquinoline | C05637 | METPA0630 |
| 2 | 0.63 | 308.0985 | 308.0982 | 1.0 | C_11_H_19_NO_9_ | [M–H]^-^ | 309.1060 | *N*-acetylneuraminic acid | C00270 | HMDB00800 |
| 3 | 0.74 | 267.0725 | 267.0729 | -1.5 | C_10_H_12_N_4_O_5_ | [M–H]^-^ | 268.0808 | Inosine | C00294 | HMDB00195 |
| 4 | 1.75 | 308.0793 | 308.0770 | 7.5 | C_14_H_15_NO_7_ | [M–H]^-^ | 309.0849 | Inodxyl glucuronide | C03033 | HMDB10319 |
| 5 | 1.92 | 144.0444 | 144.0443 | 0.7 | C_9_H_7_NO | [M–H]^-^ | 145.0528 | Indole-3-carboxaldehyde | C08493 | HMDB29737 |
| 6 | 1.98 | 178.0528 | 178.0534 | -3.4 | C_8_H_9_N_3_O_2_ | [M–H]^-^ | 179.0695 | Hippuric acid | C01586 | HMDB00714 |
| 7 | 2.45 | 146.0606 | 146.0606 | 0 | C_9_H_9_NO | [M–H]^-^ | 147.0684 | 3-methyloxindole | C02366 | HMDB04186 |
| 8 | 2.45 | 217.1039 | 217.1039 | 0 | C_12_H_14_N_2_O_2_ | [M–H]^-^ | 218.1055 | *N*-acetylserotonin | C00978 | HMDB01238 |
| 9 | 2.7 | 121.0650 | 121.0653 | -2.5 | C_8_H_10_O | [M–H]^-^ | 122.0732 | 4-methylbenzyl alcohol | C06757 | HMDB41609 |
| 10 | 3.49 | 514.2838 | 514.2838 | 0 | C_26_H_45_NO_7_S | [M–H]^-^ | 515.2917 | Taurocholic acid | C05122 | HMDB00036 |
| 11 | 4.07 | 365.2329 | 365.2328 | 0.3 | C_21_H_34_O_5_ | [M–H]^-^ | 366.2406 | Urocortisol | C05472 | HMDB00949 |
| 12 | 4.47 | 407.2777 | 407.2797 | -4.9 | C_24_H_40_O_5_ | [M–H]^-^ | 408.2876 | Cholic acid | C00695 | HMDB00619 |
| 13 | 5.18 | 391.2840 | 391.2848 | -2.0 | C_24_H_40_O_4_ | [M–H]^-^ | 392.2927 | Ursodeoxycholic acid | C07880 | HMDB00946 |
| 14 | 6.09 | 297.2426 | 297.2430 | -1.3 | C_18_H_34_O_3_ | [M–H]^-^ | 298.2508 | 12*R*-hydroxy-9*Z*-octadecenoic acid | C08365 | HMDB34297 |
| 15 | 6.64 | 455.3412 | 455.3212 | 0 | C_30_H_48_O_3_ | [M–H]^-^ | 456.3603 | Ursolic acid | C08988 | HMDB02395 |

**Table S15** MX affecting metabolite biomarkers of colorectal contents

|  | MX treatment |
| --- | --- |
| (*R*)-salsolinol | √ |
| 13*S*-hydroxyoctadecadienoic acid | √ |
| 17α-ethynylestradiol | √ |
| 3*β*,7α-dihydroxy-5-cholestenoate | √ |
| 4-(2-aminophenyl)-2,4-dioxobutanoic acid | √ |
| 7a,12a-dihydroxy-3-oxo-4-cholenoic acid | √ |
| 7α-hydroxydehydroepiandrosterone | √ |
| 9,10-DHOME | √ |
| Arachidonic acid | - |
| Cervonoylethanolamide | √ |
| Cholic acid | √ |
| Coproporphyrin III | √ |
| Dehydroepiandrosterone | √ |
| Dopamine 4-sulfate | √ |
| D-urobilinogen | √ |
| Mesobilirubinogen | √ |
| Methylimidazoleacetic acid | √ |
| Sphinganine | - |
| Cholesterol sulfate | √ |
| LysoPC (18:2(9*Z*,12*Z*)) | √ |
|  | 18 |

**Table S16** MX affecting metabolite biomarkers of fecal contents

|  |  | MX treatment |
| --- | --- | --- |
| 3-hydroxybenzoic acid |  | √ |
| 4-oxo-retinoic acid |  | - |
| 4-propylphenol |  | √ |
| 5-methoxyindoleacetate |  | √ |
| 6-hydroxynicotinic acid |  | √ |
| *N*-acetyl-L-aspartic acid |  | √ |
| *N*-acetylornithine |  | √ |
| Prenyl benzoate |  | √ |
| Spermine |  | √ |
| (*R*)-salsolinol |  | √ |
| 11*β*-hydroxyprogesterone |  | √ |
| 20-carboxy-leukotriene B4 |  | √ |
| 3-methoxy-4-hydroxyphenylglycolaldehyde | | √ |
| D-urobilinogen |  | √ |
| Hydroxyphenyllactic acid |  | √ |
| Isoeugenol |  | √ |
| Kynurenic acid |  | √ |
| Lipoxin A4 |  | √ |
| Methylphenidate |  | √ |
| *N*-formyl-L-glutamic acid |  | √ |
| Phenyllactic acid |  | √ |
| 5-L-Glutamyl-taurine |  | √ |
| α-linolenic acid |  | √ |
| Cholic acid |  | √ |
|  |  | 23 |

**Table S17** The sodium sulfate content in MX (*n* = 5)

| Batch number | 1 | 2 | 3 | 4 | 5 | Mean（%） | RSD（%） |
| --- | --- | --- | --- | --- | --- | --- | --- |
| 160504 | 99.1 | 99.3 | 99.7 | 99.0 | 99.2 | 99.26 | 0.27 |
| 170702 | 99.2 | 99.7 | 99.1 | 99.1 | 99.3 | 99.28 | 0.25 |
| 200201 | 99.3 | 99.0 | 99.0 | 99.3 | 99.5 | 99.22 | 0.21 |

**Table S18** Biological Reproducibility of Figure 1

|  | Biological replicates | | | | |
| --- | --- | --- | --- | --- | --- |
| Indicator | Con | Mod | MX-H | MX-M | MX-L |
| Number of tumors | 20 | 20 | 20 | 20 | 20 |
| Tumor diameter | 20 | 20 | 20 | 20 | 20 |
| Tumor weight | 5 | 5 | 5 | 5 | 5 |
| Tumor volume | 20 | 20 | 20 | 20 | 20 |
| Villus height | 5 | 5 | 5 | 5 | 5 |
| Crypt depth | 5 | 5 | 5 | 5 | 5 |
| Intestinal wall thinckness | 5 | 5 | 5 | 5 | 5 |
| IL-6 | 5 | 5 | 5 | 5 | 5 |
| INF-γ | 5 | 5 | 5 | 5 | 5 |
| TNF-α | 5 | 5 | 5 | 5 | 5 |
| CEA | 5 | 5 | 5 | 5 | 5 |
| CA199 | 5 | 5 | 5 | 5 | 5 |
| CA242 | 5 | 5 | 5 | 5 | 5 |

**Table S19** Biological Reproducibility of Figure 3

|  | Biological replicates | | |
| --- | --- | --- | --- |
| Indicator | Con | Mod | MX |
| α-diversity measure  Chao1 | 20 | 20 | 20 |
| The relative abundance of bacteria in Phylum level | 20 | 20 | 20 |

**Table S20**  Biological Reproducibility of Figure 4

|  | Biological replicates | | |
| --- | --- | --- | --- |
| Indicator | Con | Mod | MX |
| The relative intensity of BSH | 5 | 5 | 5 |

**Table S21** Biological Reproducibility of Figure 5

|  | Biological replicates | | |
| --- | --- | --- | --- |
| Indicator | Con | Mod | MX |
| Number of tumors | 20 | 20 | 20 |
| Tumor diameter | 20 | 20 | 20 |
| Tumor weight | 5 | 5 | 5 |
| Tumor volume | 20 | 20 | 20 |
| IL-6 | 5 | 5 | 5 |
| INF-γ | 5 | 5 | 5 |
| TNF-α | 5 | 5 | 5 |
| Villus height | 5 | 5 | 5 |
| Crypt depth | 5 | 5 | 5 |
| Intestinal wall thinckness | 5 | 5 | 5 |
| CEA | 5 | 5 | 5 |
| CA199 | 5 | 5 | 5 |
| CA242 | 5 | 5 | 5 |
| α-diversity measure Chao1 | 20 | 20 | 20 |
| The relative abundance of Lactobacillus acidophilus | 5 | 5 | 5 |

**Table S22**  Biological Reproducibility of Figure 6

|  | Biological replicates | |
| --- | --- | --- |
| Indicator | Con | MX |
| The relative abundance of bacteria in Phylum level. | 15 | 15 |

**Table S23**  Biological Reproducibility of Figure 7

|  | Biological replicates | | | |
| --- | --- | --- | --- | --- |
| Indicator | Con | Mod | MX | FexD |
| The expression of FXR | 5 | 5 | 5 |  |
| The number of tumors | 7 | 7 | 7 | 7 |
| Tumors volume | 7 | 7 | 7 | 7 |
| TBA | 7 | 7 | 7 | 7 |
| T-CHO | 7 | 7 | 7 | 7 |
| LDL-C | 7 | 7 | 7 | 7 |
| HDL-C | 7 | 7 | 7 | 7 |
| The expression of β-catenin | 7 | 7 | 7 | 7 |
| Comparison of α-diversity | 7 | 7 | 7 | 7 |
| The comparison of the Lactobacillus | 7 | 7 | 7 | 7 |

**Table S24**  Biological Reproducibility of **Figure S** 7

|  | Biological replicates | | |
| --- | --- | --- | --- |
| Indicator | Con | Mod | MX |
| The relative intensity of biomarkers | 20 | 20 | 20 |

**Table S25**  Biological Reproducibility of **Figure S** 8

|  | Biological replicates | | |
| --- | --- | --- | --- |
| Indicator | Con | Mod | MX |
| The relative intensity of biomarkers | 20 | 20 | 20 |

**Table S26**  Biological Reproducibility of **Figure S** 9

|  | Biological replicates | | |
| --- | --- | --- | --- |
| Indicator | Con | Mod | MX |
| The relative intensity of biomarkers | 20 | 20 | 20 |

**Table S27**  Biological Reproducibility of **Figure S** 10

|  | Biological replicates | | |
| --- | --- | --- | --- |
| Indicator | Con | Mod | MX |
| The relative intensity of biomarkers | 20 | 20 | 20 |

**Table S28**  Biological Reproducibility of **Figure S**15

|  | Biological replicates | |  |
| --- | --- | --- | --- |
| Indicator | Con | MX | |
| The relative intensity of metabolites | 15 | 15 | |

**Table S29 Genetic background of APC and C57BL6J ( prevention and treatment )**

| sample NO. | Position | Group |
| --- | --- | --- |
| 20 | A1 | wt/wt |
| 21 | B1 | mut/wt |
| 22 | C1 | wt/wt |
| 23 | D1 | mut/wt |
| 24 | E1 | mut/wt |
| 25 | F1 | wt/wt |
| 26 | G1 | wt/wt |
| 27 | H1 | wt/wt |
| 28 | A2 | mut/wt |
| 29 | B2 | wt/wt |
| 30 | C2 | mut/wt |
| 31 | D2 | wt/wt |
| 32 | E2 | mut/wt |
| 33 | F2 | wt/wt |
| 34 | G2 | mut/wt |
| 35 | H2 | wt/wt |
| 36 | A3 | mut/wt |
| 37 | B3 | mut/wt |
| 38 | C3 | mut/wt |
| 39 | D3 | mut/wt |
| 40 | E3 | wt/wt |
| 41 | F3 | wt/wt |
| 42 | G3 | mut/wt |
| 43 | H3 | mut/wt |
| 478 | A4 | mut/wt |
| 479 | B4 | mut/wt |
| 480 | C4 | wt/wt |
| 481 | D4 | mut/wt |
| 482 | E4 | mut/wt |
| 483 | F4 | mut/wt |
| 484 | G4 | wt/wt |
| 485 | H4 | wt/wt |
| 486 | A5 | mut/wt |
| 487 | B5 | mut/wt |
| 488 | C5 | wt/wt |
| 489 | D5 | wt/wt |
| 490 | E5 | mut/wt |
| 491 | F5 | mut/wt |
| 492 | G5 | wt/wt |
| 493 | H5 | mut/wt |
| 494 | A6 | mut/wt |
| 495 | B6 | wt/wt |
| 496 | C6 | wt/wt |
| 497 | D6 | mut/wt |
| 498 | E6 | wt/wt |
| 499 | F6 | mut/wt |
| 500 | G6 | wt/wt |
| 501 | H6 | wt/wt |
| 502 | A7 | mut/wt |
| 503 | B7 | mut/wt |
| 504 | C7 | mut/wt |
| 505 | D7 | wt/wt |
| 506 | E7 | wt/wt |
| 507 | F7 | wt/wt |
| 508 | G7 | wt/wt |
| 509 | H7 | mut/wt |
| 510 | A8 | mut/wt |
| 511 | B8 | wt/wt |
| 512 | C8 | wt/wt |
| 513 | D8 | wt/wt |
| 514 | E8 | mut/wt |
| 515 | F8 | wt/wt |
| 516 | G8 | mut/wt |
| 517 | H8 | wt/wt |
| 518 | A9 | wt/wt |
| 519 | B9 | wt/wt |
| 520 | C9 | mut/wt |
| 521 | D9 | wt/wt |
| mut/wt | E9 | mut/wt |
| wt/wt | G9 | wt/wt |
| N | H9 | Negative |
| 94 | A1 | wt/wt |
| 95 | B1 | wt/wt |
| 96 | C1 | mut/wt |
| 97 | D1 | wt/wt |
| 98 | E1 | mut/wt |
| 99 | F1 | wt/wt |
| 100 | G1 | wt/wt |
| 101 | H1 | mut/wt |
| 102 | A2 | wt/wt |
| 103 | B2 | mut/wt |
| 104 | C2 | wt/wt |
| 105 | D2 | wt/wt |
| 106 | E2 | wt/wt |
| 107 | F2 | mut/wt |
| 108 | G2 | mut/wt |
| 109 | H2 | mut/wt |
| 110 | A3 | wt/wt |
| 111 | B3 | wt/wt |
| 112 | C3 | wt/wt |
| 113 | D3 | wt/wt |
| 114 | E3 | mut/wt |
| 115 | F3 | mut/wt |
| 116 | G3 | wt/wt |
| 117 | H3 | wt/wt |
| 118 | A4 | mut/wt |
| 119 | B4 | wt/wt |
| 120 | C4 | wt/wt |
| 121 | D4 | mut/wt |
| 122 | E4 | wt/wt |
| 123 | F4 | wt/wt |
| 124 | G4 | wt/wt |
| 125 | H4 | wt/wt |
| 126 | A5 | mut/wt |
| 127 | B5 | wt/wt |
| 128 | C5 | mut/wt |
| 129 | D5 | mut/wt |
| 130 | E5 | wt/wt |
| 131 | F5 | wt/wt |
| 132 | G5 | wt/wt |
| 133 | H5 | wt/wt |
| 134 | A6 | mut/wt |
| 135 | B6 | wt/wt |
| 136 | C6 | wt/wt |
| 137 | D6 | wt/wt |
| 138 | E6 | mut/wt |
| 139 | F6 | mut/wt |
| 140 | G6 | wt/wt |
| 141 | H6 | mut/wt |
| 142 | A7 | wt/wt |
| 143 | B7 | mut/wt |
| 144 | C7 | mut/wt |
| 145 | D7 | wt/wt |
| 146 | E7 | mut/wt |
| 147 | F7 | wt/wt |
| 148 | G7 | wt/wt |
| 149 | H7 | mut/wt |
| 150 | A8 | wt/wt |
| 151 | B8 | wt/wt |
| 152 | C8 | mut/wt |
| 153 | D8 | wt/wt |
| 154 | E8 | wt/wt |
| 155 | F8 | mut/wt |
| 156 | G8 | wt/wt |
| 157 | H8 | mut/wt |
| 158 | A9 | mut/wt |
| 159 | B9 | wt/wt |
| 160 | C9 | wt/wt |
| 161 | D9 | wt/wt |
| 162 | E9 | wt/wt |
| 163 | F9 | mut/wt |
| 164 | G9 | mut/wt |
| 165 | H9 | wt/wt |
| 166 | A10 | wt/wt |
| 167 | B10 | mut/wt |
| 168 | C10 | mut/wt |
| 169 | D10 | wt/wt |
| 170 | E10 | wt/wt |
| 171 | F10 | wt/wt |
| 172 | G10 | wt/wt |
| 173 | H10 | mut/wt |
| 174 | A11 | mut/wt |
| 175 | B11 | wt/wt |
| 176 | C11 | mut/wt |
| 177 | D11 | mut/wt |
| 178 | E11 | mut/wt |
| 179 | F11 | wt/wt |
| 180 | G11 | mut/wt |
| 181 | H11 | wt/wt |
| mut/wt | A12 | mut/wt |
| wt/wt | C12 | wt/wt |
| N | D12 | Negative |

**Table S29** Genetic background of APC and C57BL6J (MX regulated Lactobacillus-bile acid-intestinal FXR axis )

| Sample NO | Position | Result |
| --- | --- | --- |
| 597 | A1 | wt/wt |
| 598 | C1 | KI/wt |
| 599 | E1 | KI/wt |
| 600 | G1 | wt/wt |
| 601 | I1 | wt/wt |
| 602 | K1 | wt/wt |
| 603 | M1 | wt/wt |
| 604 | O1 | wt/wt |
| 605 | A3 | wt/wt |
| 606 | C3 | KI/wt |
| 607 | E3 | wt/wt |
| 608 | G3 | wt/wt |
| 609 | I3 | wt/wt |
| 610 | K3 | wt/wt |
| 611 | M3 | KI/wt |
| 612 | O3 | KI/wt |
| 613 | A5 | KI/wt |
| 614 | C5 | wt/wt |
| 615 | E5 | wt/wt |
| 616 | G5 | wt/wt |
| 617 | I5 | wt/wt |
| 618 | K5 | wt/wt |
| 619 | M5 | wt/wt |
| 620 | O5 | wt/wt |
| 621 | A7 | wt/wt |
| 622 | C7 | wt/wt |
| 623 | E7 | KI/wt |
| 624 | G7 | KI/wt |
| 625 | I7 | wt/wt |
| 626 | K7 | KI/wt |
| 627 | M7 | wt/wt |
| 628 | O7 | wt/wt |
| 629 | A9 | wt/wt |
| 630 | C9 | wt/wt |
| 631 | E9 | KI/wt |
| 632 | G9 | wt/wt |
| 633 | I9 | wt/wt |
| 634 | K9 | wt/wt |
| 635 | M9 | wt/wt |
| 636 | O9 | KI/wt |
| 637 | A11 | KI/wt |
| 638 | C11 | wt/wt |
| 639 | E11 | wt/wt |
| 640 | G11 | KI/wt |
| 641 | I11 | wt/wt |
| 642 | K11 | KI/wt |
| 643 | M11 | wt/wt |
| 644 | O11 | wt/wt |
| 645 | A13 | KI/wt |
| 646 | C13 | KI/wt |
| 647 | E13 | KI/wt |
| 648 | G13 | KI/wt |
| 649 | I13 | wt/wt |
| 650 | K13 | wt/wt |
| 651 | M13 | KI/wt |
| 652 | O13 | KI/wt |
| 653 | A15 | KI/wt |
| 654 | C15 | wt/wt |
| 655 | E15 | wt/wt |
| 656 | G15 | KI/wt |
| 657 | I15 | wt/wt |
| 658 | K15 | KI/wt |
| 659 | M15 | wt/wt |
| 660 | O15 | wt/wt |
| 661 | A17 | KI/wt |
| 662 | C17 | KI/wt |
| 663 | E17 | wt/wt |
| 664 | G17 | KI/wt |
| 665 | I17 | wt/wt |
| 666 | K17 | KI/wt |
| 667 | M17 | KI/wt |
| 668 | O17 | wt/wt |
| 669 | A19 | KI/wt |
| 670 | C19 | KI/wt |
| 671 | E19 | wt/wt |
| 672 | G19 | wt/wt |
| 673 | I19 | wt/wt |
| 674 | K19 | wt/wt |
| 675 | M19 | KI/wt |
| 676 | O19 | KI/wt |
| 677 | A21 | wt/wt |
| 678 | C21 | wt/wt |
| 679 | E21 | wt/wt |
| 680 | G21 | KI/wt |
| 681 | I21 | KI/wt |
| 682 | K21 | KI/wt |
| 683 | M21 | KI/wt |
| 684 | O21 | wt/wt |
| 685 | A23 | wt/wt |
| 686 | C23 | KI/wt |
| 687 | E23 | wt/wt |
| 688 | G23 | wt/wt |
| 689 | I23 | KI/wt |
| 690 | K23 | KI/wt |
| 691 | M23 | KI/wt |
| 692 | O23 | wt/wt |
| 513 | B1 | wt/wt |
| 514 | D1 | wt/wt |
| 515 | F1 | KI/wt |
| 516 | H1 | wt/wt |
| 517 | J1 | wt/wt |
| 518 | L1 | wt/wt |
| 519 | N1 | KI/wt |
| 520 | P1 | KI/wt |
| 521 | B3 | wt/wt |
| 522 | D3 | wt/wt |
| 523 | F3 | KI/wt |
| 524 | H3 | wt/wt |
| 525 | J3 | KI/wt |
| 526 | L3 | KI/wt |
| 527 | N3 | wt/wt |
| 528 | P3 | KI/wt |
| 529 | B5 | wt/wt |
| 530 | D5 | wt/wt |
| 531 | F5 | KI/wt |
| 532 | H5 | KI/wt |
| 533 | J5 | wt/wt |
| 534 | L5 | KI/wt |
| 535 | N5 | KI/wt |
| 536 | P5 | KI/wt |
| 537 | B7 | wt/wt |
| 538 | D7 | KI/wt |
| 539 | F7 | wt/wt |
| 540 | H7 | wt/wt |
| 541 | J7 | KI/wt |
| 542 | L7 | wt/wt |
| 543 | N7 | KI/wt |
| 544 | P7 | wt/wt |
| 545 | B9 | wt/wt |
| 546 | D9 | KI/wt |
| 547 | F9 | KI/wt |
| 548 | H9 | wt/wt |
| 549 | J9 | KI/wt |
| 550 | L9 | wt/wt |
| 551 | N9 | wt/wt |
| 552 | P9 | KI/wt |
| 553 | B11 | wt/wt |
| 554 | D11 | KI/wt |
| 555 | F11 | KI/wt |
| 556 | H11 | KI/wt |
| 557 | J11 | wt/wt |
| 558 | L11 | wt/wt |
| 559 | N11 | KI/wt |
| 560 | P11 | wt/wt |
| 561 | B13 | wt/wt |
| 562 | D13 | wt/wt |
| 563 | F13 | wt/wt |
| 564 | H13 | KI/wt |
| 565 | J13 | wt/wt |
| 566 | L13 | KI/wt |
| 567 | N13 | KI/wt |
| 568 | P13 | wt/wt |
| 569 | B15 | wt/wt |
| 570 | D15 | KI/wt |
| 571 | F15 | KI/wt |
| 572 | H15 | KI/wt |
| 573 | J15 | KI/wt |
| 574 | L15 | KI/wt |
| 575 | N15 | wt/wt |
| 576 | P15 | wt/wt |
| 577 | B17 | KI/wt |
| 578 | D17 | wt/wt |
| 579 | F17 | KI/wt |
| 580 | H17 | KI/wt |
| 581 | J17 | KI/wt |
| 582 | L17 | KI/wt |
| 583 | N17 | KI/wt |
| 584 | P17 | wt/wt |
| 585 | B19 | KI/wt |
| 586 | D19 | wt/wt |
| 587 | F19 | KI/wt |
| 588 | H19 | wt/wt |
| 589 | J19 | wt/wt |
| 590 | L19 | KI/wt |
| 591 | N19 | wt/wt |
| 592 | P19 | wt/wt |
| 593 | B21 | KI/wt |
| 594 | D21 | wt/wt |
| 595 | F21 | KI/wt |
| 596 | H21 | KI/wt |
| 693 | A2 | wt/wt |
| 694 | C2 | KI/wt |
| 695 | E2 | wt/wt |
| 696 | G2 | wt/wt |
| 697 | I2 | KI/wt |
| 698 | K2 | wt/wt |
| 699 | M2 | KI/wt |
| 700 | O2 | KI/wt |
| 701 | A4 | KI/wt |
| 702 | C4 | KI/wt |
| 703 | E4 | wt/wt |
| 704 | G4 | wt/wt |
| 705 | I4 | wt/wt |
| 706 | K4 | KI/wt |
| 707 | M4 | KI/wt |
| 708 | O4 | wt/wt |
| 709 | A6 | KI/wt |
| 710 | C6 | wt/wt |
| 711 | E6 | wt/wt |
| 712 | G6 | KI/wt |
| 713 | I6 | wt/wt |
| 714 | K6 | KI/wt |
| 715 | M6 | wt/wt |
| 716 | O6 | wt/wt |
| 717 | A8 | KI/wt |
| 718 | C8 | KI/wt |
| 719 | E8 | KI/wt |
| 720 | G8 | wt/wt |
| 721 | I8 | wt/wt |
| 722 | K8 | KI/wt |
| 723 | M8 | wt/wt |
| 724 | O8 | wt/wt |
| 725 | A10 | KI/wt |
| 726 | C10 | wt/wt |
| 727 | E10 | KI/wt |
| 728 | G10 | KI/wt |
| 729 | I10 | wt/wt |
| 730 | K10 | wt/wt |
| 731 | M10 | KI/wt |
| 732 | O10 | wt/wt |
| 733 | A12 | wt/wt |
| 734 | C12 | KI/wt |
| 735 | E12 | KI/wt |
| 736 | G12 | wt/wt |
| 737 | I12 | KI/wt |
| 738 | K12 | KI/wt |
| 739 | M12 | wt/wt |
| 740 | O12 | KI/wt |
| 741 | A14 | wt/wt |
| 742 | C14 | wt/wt |
| 743 | E14 | KI/wt |
| 744 | G14 | KI/wt |
| 745 | I14 | KI/wt |
| 746 | K14 | KI/wt |
| 747 | M14 | KI/wt |
| 748 | O14 | KI/wt |
